# IntEREst: intron-exon retention estimator

**DOI:** 10.1186/s12859-018-2122-5

**Published:** 2018-04-11

**Authors:** Ali Oghabian, Dario Greco, Mikko J. Frilander

**Affiliations:** 1 Institute of Biotechnology, University of Helsinki, P.O. Box 56 (Viikinkaari 5), FI-00014 Helsinki, Finland; 2 Faculty of Medicine and Life Sciences, Tampere, Finland; 3 Institute of Biosciences and Medical Technologies (BioMediTech), Arvo Ylpön Katu 34, FI-33014 University of Tampere, Tampere, Finland

**Keywords:** RNA-seq, Intron retention, Alternative splicing, RNA, Expression analysis, Bioconductor, U12-type introns, U2-type introns

## Abstract

**Background:**

In-depth study of the intron retention levels of transcripts provide insights on the mechanisms regulating pre-mRNA splicing efficiency. Additionally, detailed analysis of retained introns can link these introns to post-transcriptional regulation or identify aberrant splicing events in human diseases.

**Results:**

We present IntEREst, Intron–Exon Retention Estimator, an R package that supports rigorous analysis of non-annotated intron retention events (in addition to the ones annotated by RefSeq or similar databases), and support intra-sample in addition to inter-sample comparisons. It accepts binary sequence alignment/map (.bam) files as input and determines genome-wide estimates of intron retention or exon-exon junction levels. Moreover, it includes functions for comparing subsets of user-defined introns (*e.g.* U12-type vs U2-type) and its plotting functions allow visualization of the distribution of the retention levels of the introns. Statistical methods are adapted from the DESeq2, edgeR and DEXSeq R packages to extract the significantly more or less retained introns. Analyses can be performed either sequentially (on single core) or in parallel (on multiple cores). We used IntEREst to investigate the U12- and U2-type intron retention in human and plant RNAseq dataset with defects in the U12-dependent spliceosome due to mutations in the ZRSR2 component of this spliceosome. Additionally, we compared the retained introns discovered by IntEREst with that of other methods and studies.

**Conclusion:**

IntEREst is an R package for Intron retention and exon-exon junction levels analysis of RNA-seq data. Both the human and plant analyses show that the U12-type introns are retained at higher level compared to the U2-type introns already in the control samples, but the retention is exacerbated in patient or plant samples carrying a mutated *ZRSR2* gene. Intron retention events caused by *ZRSR2* mutation that we discovered using IntEREst (DESeq2 based function) show considerable overlap with the retained introns discovered by other methods (*e.g.* IRFinder and edgeR based function of IntEREst). Our results indicate that increase in both the number of biological replicates and the depth of sequencing library promote the discovery of retained introns, but the effect of library size gradually decreases with more than 35 million reads mapped to the introns.

**Electronic supplementary material:**

The online version of this article (10.1186/s12859-018-2122-5) contains supplementary material, which is available to authorized users.

## Background

Alternative pre-mRNA splicing is a cellular process in eukaryotes that generates multiple transcripts from a single gene. Of the various types of alternative splicing (reviewed by Hamid and Makeyev [1]) intron retention (IR) events have been less characterized than the alternative splicing events that are more frequent in mammals, such as exon skipping and choice of alternative 5' splice site (5'ss) and 3' splice site (3'ss). While the best characterized IR events have been detected from humans with diseases caused by mutations in the core pre-mRNA splicing machinery, recent work has established that regulated IR events are also part of the normal regulation of gene expression [2, 3] and function in important biological processes such as cellular differentiation [4]. Furthermore, in some taxa such as plants, IR is one of the most prominent mechanisms of alternative splicing [5].

A well-established example of IR involves U12-type introns (also called minor introns), which are spliced less efficiently compared to the U2-type (major) introns [6]. The classification to major U2-type introns and minor U12-type introns derives from the coexistence of two parallel pre-mRNA splicing machineries in the cells of most metazoan species. Majority of metazoan introns are excised by the "major" U2-dependent spliceosome, and are therefore referred as the U2-type or major introns. A small subset of metazoan introns, approximately 0.35% or roughly 700-800 introns in mammals, are excised by a parallel U12-dependent spliceosome, also known as the minor spliceosome [7]. The targets of the U12-dependent spliceosome are minor introns, which feature highly conserved, but divergent 5'ss and branch point sequences (BPS), which makes it possible to identify these introns computationally [8, 9]. One of the main characteristics of the minor spliceosome is that it is less efficient compared to the major spliceosome [10–12]. As a result of the inefficient splicing, elevated levels of transcripts containing unspliced minor introns are retained in the nucleus and targeted by nuclear RNA decay pathways [6]. Moreover, disease-causing mutations in the snRNA and protein components of the minor spliceosome (*e.g.* U4atac and U12 snRNAs, U11/U12-65K and ZRSR2 proteins) show, among other splicing defects, a further increase in IR levels of the U12-type introns [13–19].

Various alternative splicing analysis tools have been developed [20–22], however few tools exist that focus on extracting novel intron retention (IR) events and perform differential IR analysis [23]. For a robust analysis of retention levels of introns within and between various samples we developed IntEREst, *i.e.* Intron–Exon Retention Estimator that is based on the intron retention analysis used in Niemelä et al. [6]. IntEREst accepts standard binary sequence alignment/map (.bam) files as an input and estimates the genome-wide retention levels of the introns using sequencing reads mapping to introns, intron-exon boundaries, or to exon-exon junctions. The results are provided both as IR fold changes and relative PSI or Ψ (percent spliced in) [24] values and can be further analyzed by any of the several statistical packages included, *e.g.* differential intron retention test based on the “exon usage test” provided by DEXSeq [25, 26], differential IR test based on count data differential analysis tools provided by DESeq2 [27], or exact test, generalized linear models and quasi-likelihood test adapted from edgeR [28, 29]. The statistical tests calculate *p*-values based on the null hypothesis that IR does not vary across the analyzed sample groups. The resulting *p*-values estimated for each intron allow subsequent identification of introns that show statistically significant difference of IR between the sample groups. IntEREst also provides tools for plotting the distribution of retention levels of the introns of interest within single or multiple samples. In addition, large datasets that demand significant computation time can be analyzed in parallel on multiple computing cores. IntEREst is available as a Bioconductor package and together with the manuals are accessible through https://bioconductor.org/packages/release/bioc/html/IntEREst.html.

### Implementation

IntEREst is an R package that supports various functions to measure the retention levels of the introns, perform statistical differential intron retention analysis across various samples, and plot the distribution of retention levels of different types of introns across various samples. The main design aim of IntEREst has been to support analysis of relative low level IR values (>10%) that are more challenging to implement with the existing software [24] but are typical for the U12-type introns [6] and for U2-type introns in human diseases with a mild defects with spliceosome function. In such cases the commonly used Ψ values, particularly with default cutoffs, may underestimate the extent of IR. Specifically, the advantages of IntEREs are the ability to use multiple test samples and controls, possibility to define complicated design experiments (incorporating various sample annotations such as age, sex, and etc.) for IR comparisons across samples, parallelization of the computation and running on multiple nodes/cores, integration to Bioconductor environment and the use of both intronic and exon junctions reads, either alone or together, to estimate the IR levels. Additionally, besides providing a global IR analysis, IntEREst supports analysis of user-defined subset of introns, *e.g*. U12-type and U2-type introns.

The RNAseq read summarization functions (*i.e. interest()* and *interest.sequential()*) accept a *.bam* read alignment file and a reference as inputs, and output the raw (un-normalized) and normalized number of fragments mapping to each exon or intron. The reference includes coordinates of exons and introns together with their annotations, such as gene and transcript names, and intron type identifier. The reference can be built using the *referencePrepare()* function supported by IntEREst. Note that the intron identifiers used in our analysis are U12- and U2-type introns, but the application of IntEREst is not limited to the comparison of these intron types. Other classifications can be defined by the user and the retention levels of the introns can be plotted and compared across the user-defined classes. The functions in the IntEREst package that are specific to the comparisons of the U2- vs U12-type introns, *e.g. u12Boxplot()*, *u12DensityPlot()*, *u12Index()* start with “u12”.

IntEREst features two functions that estimate the raw and normalized intron retention levels: 1) *interest()*, capable of running in parallel on multiple computing cores and 2*) interest.sequential()*, that runs sequentially on a single computing core. These functions use the *bpiterate()* function from the BiocParallel R Bioconductor package [30] to read and analyze the mapped reads, *m* reads at a time (by default *m* = 1 million) to comply with the limitations of the memory usage in the running environment. When running *interest.sequential()*, the mapped reads are analyzed as batches of *m* reads (or read pairs if the *isPaired* parameter is set TRUE) at a time on a single computing core. With *interest()* it is possible to analyze *n* batches of *m* reads (*i.e. m×n* reads or read pairs) simultaneously while they are distributed over *n* computing cores and repeat this process until all reads have been analyzed.

The summarization functions *interest.sequential()* and *interest()* support two distinct analysis modes: 1) intron-exon junction estimation and 2) exon-exon junction estimation. It is possible to configure the analysis to include only the reads that map to intron-exon or exon-exon junctions, however with default settings reads that map entirely to the intronic or exonic regions are also included in the calculation of retention level estimates. For a typical intron-exon junction estimation analysis, we recommend to collapse the overlapping exonic coordinates across various splicing isoforms in the reference to avoid any biases in the IR calculation that may be introduced by the read counts of alternative exons, or by exonic regions overlapping with sequences annotated as introns in other transcripts. To improve the running time and avoid repetitive processes, in exon-exon junction analysis mode, we recommend using a filtered reference resulting from the *unionRefTr()* function. This function identifies all repeating exons and uses only a single copy of each. Moreover, because repetitive sequence elements may bias the read mapping and thus affect the IR estimates, the read summarization functions support the possibility to exclude the repeat regions and reads that map to such regions. The default normalization method applied in the read summarization functions is Fragments Per Kilobase per Million mapped fragments (FPKM) however, it is scaled at transcript level (formula 1). For every intron *i* of gene *g* with *I* introns, if the length of the intron is *L*_*ig*_ and the number of fragments mapped to the intron is *X*_*ig*_ its normalized retention value will be *FPKM*_*ig*_:

1 $$ {FPKM}_{ig}=\frac{X_{ig}}{L_{ig}\cdot \sum \limits_{k=1}^I{X}_{kg}}\times {10}^9 $$

IntEREst provides a function *lfc()* that estimates the log_2_ FC of the retention levels across two various conditions, moreover it includes a function *psi()* to measure the Ψ values, i.e. the percent spliced in, for all studied introns. We have adapted several statistical tests from multiple sources for intron retention and exon-junctions analysis: DESEq2 [27], edgeR [21, 22], and DEXSeq [25, 26]. All these methods can be used to study the intron retention changes across the samples in a genome-wide scale. However, the DEXSeq based method (*i.e. DEXSeqInterest()* function) differs from the others as it uses the differential exon usage method to perform gene-wise comparisons

## Results and discussion

### Genome-wide analysis of retention of U2 and U12-type introns

To demonstrate the application of IntEREst in comparing retention levels of various types of introns across several samples, we reanalyzed the RNAseq data from myelodysplastic syndrome (MDS) patients and control subjects included in Madan et al. [17] study. Specifically, we compared the genome-wide retention levels of U12-type introns vs U2-type across the MDS samples. This disease is caused by mutations in the *ZRSR2* gene that encodes an integral protein component of the minor spliceosome. Moreover, the original analysis of the dataset reported that the *ZRSR2* mutations in the patient samples led to increased retention of primarily U12-type introns while the U2-type introns were reported to be less affected [17]. The dataset represents 16 individuals: 8 were diagnosed with MDS and featured mutations in the *ZRSR2* gene (referred to as ZRSR2mut), 4 were diagnosed with MDS but lacked the *ZRSR2* mutations (referred to as ZRSR2wt), and 4 were healthy individuals (HEALTHY).

We ran genome-wide retention comparison of U12-type introns to U2-type introns. To carry out the analysis, we used RefSeq as a reference and identified and annotated 510 U12-type introns using the *annotateU12()* function that uses Position Weigh Matrices (PWM) extracted from the U12DB database [9]. Next we performed the differential IR analysis using the DESeq2-based function of IntEREst (comparing the ZRSR2mut samples vs ZRSR2wt and HEALTHY). The DESeq2 test was run by considering both results from intron retention and exon-exon junction runs of *interest()* function. Initially, by using the *interestResultIntEx()* function a result object was built that includes information of both intron retention and exon-exon junction levels (see Additional file 1 for more details).

**Fig. 1:**
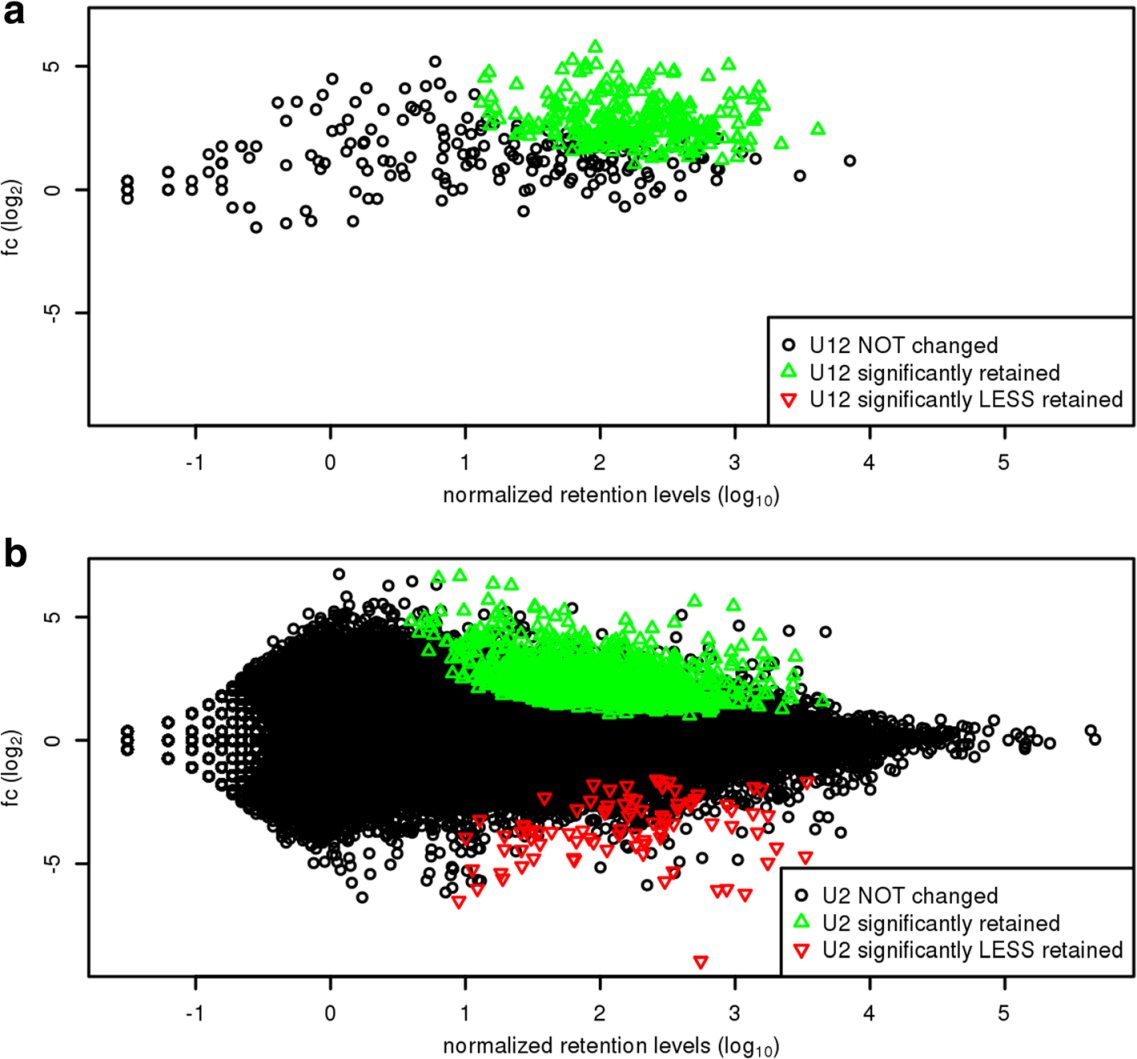
Intron retention level distribution. Retention log2 fold change (ZRSR2mut vs control) vs normalized retention levels of U12-type introns (**a**) and U2-type introns (**b**). Introns showing significantly higher and lower IR values have been indicated with green and red color, respectively

**Fig. 2:**
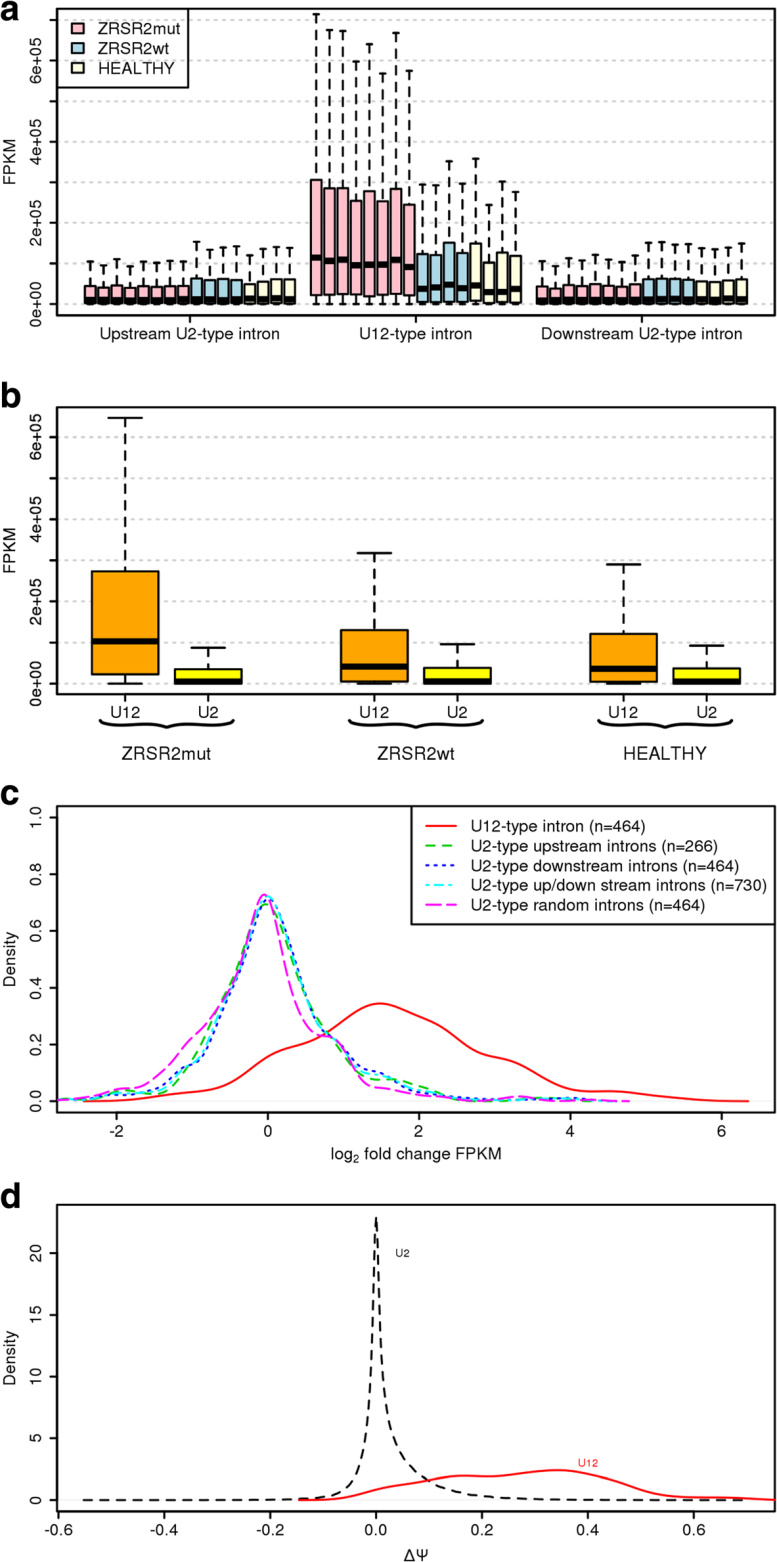
FPKM-scaled retention levels of U12-type and U2-type introns across various samples in MDS data, excluding transcripts that feature only introns with low average read counts over all samples (i.e. 1 read or less). **a** Boxplot showing FPKM-scaled retention levels of the U12-type introns (middle) as compared to their upstream and downstream U2-type intron. The thick horizontal lines in boxplots represents the median values and the whiskers represent 1.5 times the interquartile range. The box extends from the first quartile to the third quartile. **b** Boxplot showing the distribution of the FPKM-scaled retention levels of U12-type introns compared to the U2-type intron in ZRSR2mut, ZRSR2wt, and HEALTHY samples. **c** Density plot illustrating the frequency of the fold change (log2) of the retention levels of U12-type introns, randomly picked U2-type introns, U2-type introns upstream and downstream of the U12-type introns when comparing ZRSR2mut to the control samples of the MDS data. **d** Density plot illustrating the frequency of the ΔΨ values (increase of percentage spliced in) of the U12- and U2-type introns when comparing ZRSR2mut to the control samples. The Ψ values are between -1 and 1

The results show an increased retention of U12-type introns in the ZRSR2mut samples as opposed to U2-type introns. Specifically, after the low retention filtering and using a 0.01 adjusted *p*-value cutoff on the DESeq2 results, we identified 1521 introns representing either the U12- or U2- type that displayed higher retention levels in the ZRSR2mut samples compared to the controls (*i.e.* ZRSR2wt and HEALTHY samples). Of the 510 U12-type introns in the data, 269 (*i.e.* 52.7% of the U12-type introns) showed significant up-regulation of IR in the ZRSR2mut samples when compared to the controls, while none of the U12-type introns showed a significant reduction in IR (see Fig. 1a). In contrast, only 1252 of the 228524 (~0.54%) of U2-type introns analyzed showed a significant increase of IR and 89 (~0.03%) showed a significant decrease (see Fig. 1b). Our analysis also confirmed the earlier observation of increased intron retention levels with U12-type introns compared to U2-type introns [6, 11, 31] since we observed that the overall FPKM retention values (formula 1) of U12-type introns were higher than that of U2-type in all the samples of the MDS study, including the ZRSR2mut, ZRSR2wt and HEALTHY samples (Fig. 2a, b). However, this effect was more prominent in the ZRSR2mut samples, suggesting that the *ZRSR2* mutations were exacerbating the IR of the U12-type introns. Similar increase in IR was not observed with the U2-type introns between ZRSR2mut and controls, regardless of whether they were located in the genes containing U12-type introns or other genes, or in the close proximity of U12-type introns (immediately up- or downstream position). Rather, the median log_2_ fold-change of the U2-type introns was approximately zero whereas the median log_2_ fold-change of U12-type introns was ~1.5 (see Fig. 2c). Moreover, the Jonckheere Trend test [32, 33] with 10000 number of permutations, under the null hypothesis that the values are similar (and with the alternative that the values for the U12-type introns are higher) returned a highly significant *p*-value of 0.0001. In line with these results, the median of ΔΨ values (i.e. the increase of percentage spliced in when comparing ZRSR2mut samples to the controls) for all U12-type introns was about 1% as compared to 0.6% with U2-type introns (see Fig. 2d). Moreover, the average ΔΨ values for introns showing a significant increase in IR were ~33% and 23% for U12-type and U2-type introns, respectively.

To further evaluate the validity and generality of our results, we compared the MDS results to the similar results that we obtained from analyzing an additional Maize data [34] (see Additional file 1 for more details). The Maize data is constructed of 6 samples (i.e. 3 roots and 3 shoots referred to as RGH3mut) that feature mutations in the gene *RGH3* (ortholog of Human *ZRSR2* gene) and 6 samples (3 roots and 3 shoots referred to as RGH3wt) that lack the mutation. The results of the Maize data analysis mirror our findings with the MDS data. Analogous to MDS data, the RGH3mut samples showed increased IR with ~46% of U12-type introns, while only a ~0.46% of the U2-type introns showed an increase in IR (see Additional file 1: Figure S7).

Together, our results suggest that IntEREst provides reliable quantification of differential IR events; Specifically, our results are not only consistent with the well-documented increased retention levels of U12-type introns [6, 11, 31], but are also in concordance with the molecular function of the ZRSR2 protein (and its Maize ortholog, *i.e.* RGH3) in the recognition of U12-type introns [17, 34].

### Benchmarking and comparison to other methods

We evaluated the performance of the IntEREst in two ways using the MDS benchmark dataset. First, we carried out internal analysis comparing IntEREst results in conjunction with different statistical analysis packages implemented in IntEREst. Subsequently, we carried out comparison with both, the published results of the MDS analysis [17] and IRfinder [23], *i.e.* dedicated software for IR analysis. Note that all comparisons described in the following are based on the introns that were available in the both references used by the compared counterparts.

### Differential up- and down–regulated introns in methods implemented in IntEREst

We compared the three methods implemented in IntEREst for differential intron retention analysis, *i.e.* DESeq2, GLM function of edgeR and DEXSeq, referred hereafter as IntEREst-DESeq2, IntEREst-edgeR and IntEREst-DEXSeq, respectively. The DESeq2 and edgeR have been previously reported to result in somewhat dissimilar results in differential gene expression analysis [35]. In contrast, DEXseq method differs in its application (see above). For IntEREst-DESeq2 and IntEREst-edgeR comparison, we first merged the intron-exon and the exon-exon junction results (obtained by running *interest()* in its two running modes) using *interestResultIntEx()*. Subsequently, we used *deseqInterest()* and *glmInterest()* functions (*i.e.* the IntEREst functions based on DESeq2 and edgeR-GLM) to analyze the change of IR relative to the change of the junction levels of their flanking exons. We used an adjusted *p*-value (Benjamini and Hochberg [36]) threshold cutoff of 0.01 to identify introns that are retained at significantly higher or lower level in the ZRSR2mut samples compared to controls (see Additional file 1 for more details).

**Fig. 3:**
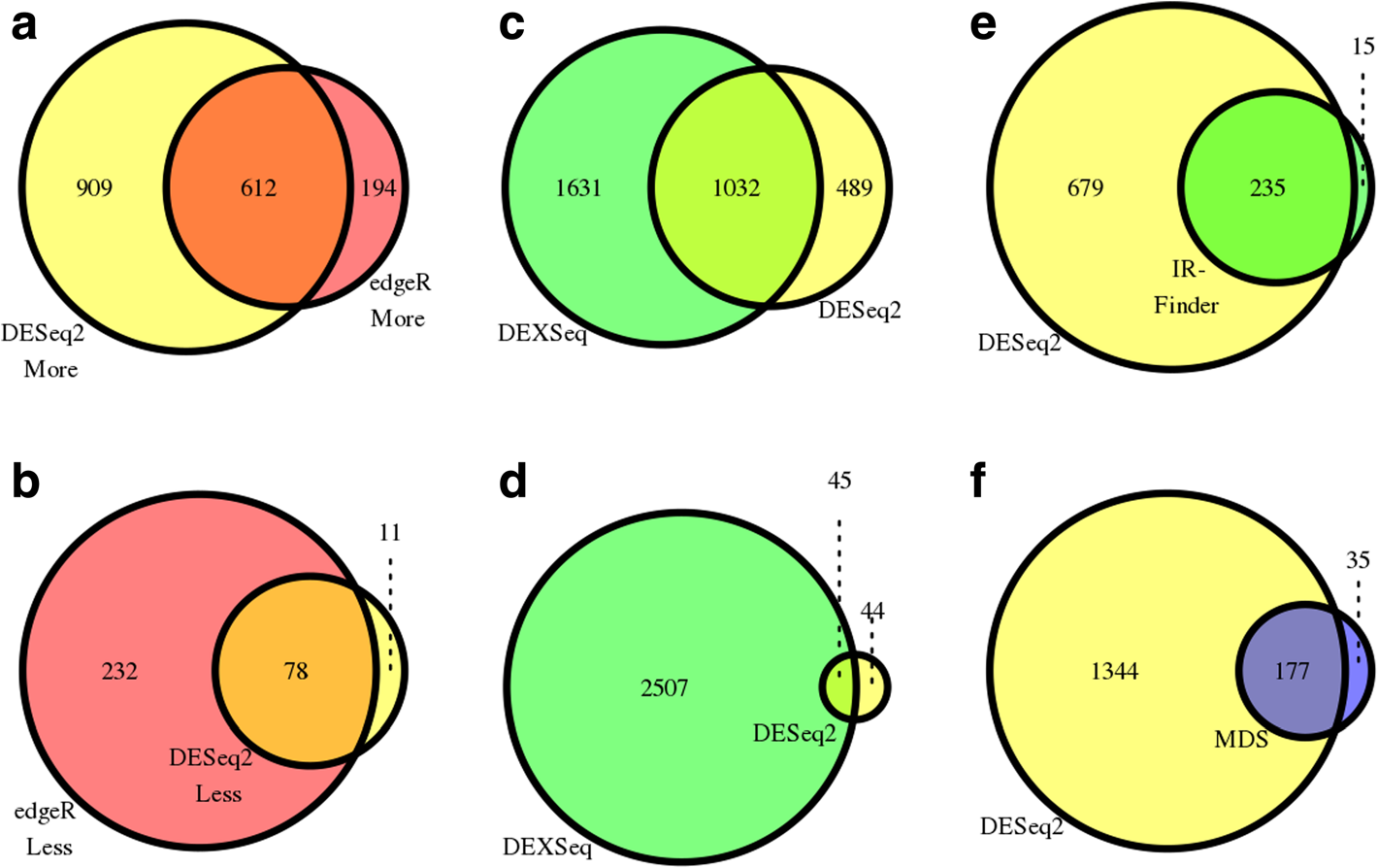
Venn diagrams showing the comparisons of: (**a**) Significantly more retained introns (labeled with “up”) discovered by Interest-DESeq2 and Interest-edgeR (**b**) As in panel A, but showing data for the significantly less retained introns (labeled with “down”). (**c**) Significantly more retained introns using the IntEREst-DESeq2 and IntEREst-DEXeq (**d**) As in panel C, but showing the significantly less retained introns. (**e**) Significantly retained introns discovered by IntEREst-DESeq2 and the IRFinder [23]. (**f**) Significantly retained introns discovered by IntEREst-DESeq2 and the significantly retained introns reported by Madan et al. [17], labeled with “MDS”. All the significant more/less retained introns were extracted from the unfiltered MDS data, comparing the ZRSR2mut to the control samples

We found that there is a significant overlap both with upregulated and downregulated introns between the IntEREst-DESeq2 and IntEREst-edgeR (Fig 3a, b), with a bias towards upregulated introns. Furthermore, of the introns not shared between the two methods, the IntEREst-DESeq2 identified more introns with an increase in IR, while the IntEREst-edgeR identified more downregulated IR events. The majority of the IR events not shared by the two methods (specifically those discovered by IntEREst-DESeq2 and missed by IntEREst-edgeR) display a weaker IR fold-change compared to those in the shared intron group (See Additional file 1: Figure S3). The observed differences between the two methods are in line with the recent DEG analysis results [35] and are due to the variability of the methods used and an extra filtering step based on Cook’s distance which is used in DESeq2 by default.

Comparison of the IntEREst-DESeq2 to IntEREst-DEXSeq revealed a considerable overlap between the two methods (Fig. 3 c). However, IntEREst-DEXSeq identified a large number of significantly less retained introns not identified by the IntEREst-DESeq2 (Fig. 3 d). This outcome reflects the gene-wise method adapted in DEXSeq where the variation in the retention levels of each intron is compared to the relative retention variation of all other introns within the same gene, rather than solely comparing the genome-wide changes of IR levels. This results in a more symmetric distribution of up/down regulated intron retention signals (Fig. S4). As a consequence, the significantly more and less retained introns discovered by IntEREst-DEXSeq were more than twice more frequently observed in the same genes compared to those identified by IntEREst-DESeq2. Furthermore, the IntEREst-DEXSeq only consider the reads that map to either introns or exons (here the intron read counts were used) and does not support the usage of both intron retention and exon-exon junction information.

### IntEREst-DESeq2 and IRFinder show extensive overlap

We next compared the IntEREst-DESeq2 to IRFinder, a dedicated IR analysis software, which also uses DESeq2 package in its downstream analysis [27]. Since IntEREst-DESeq2 counts reads that map to the exons, we used the mean of the number of reads mapping to the 5’ and 3’ flanking exons. In contrast, the IRFinder counts the junction reads that map across the flanking exons. Running IRFinder with the default parameters extracted 250 introns showing significantly increased IR in ZRSR2mut samples, most of which (*i.e.* 235) overlapped with the introns discovered by the IntEREst-DESeq2 (Fig. 3 e). Note that IntEREst utilized more intron/exon-mapped reads compared to IRFinder. This was particularly evident with introns with lower retention levels, thus providing better-supported fold-change estimates for such introns (Additional file 1: Figure S5).

### Enhanced discovery of IR events in MDS samples

We further compared our IR results with the original analysis of the MDS dataset by Madan et al. [17]. We found that IntEREs-DESeq2 was able to identify most (*i.e.* 177 out of 205 introns) of the significant IR events reported by Madan et al. [17], but it also discovered a large number of additional events not reported in the original study (Fig. 3f), representing both U12-type (149) and U2-type (1195) introns. On the contrary, the events that were reported in the original study, but missed in our analysis all represent borderline cases featuring low fold-changes and statistical significance (Additional file 1: Figure S6).

Together, our results revealed that the different methods implemented in IntEREst are able to identify a highly overlapping set of high-confidence differentially retained introns. Additionally, each method also identified IR events that are unique to a particular method. This provides the flexibility to select an approach best fitting to the particular research questions.

### Sample size and sequencing library size sensitivity

**Fig. 4:**
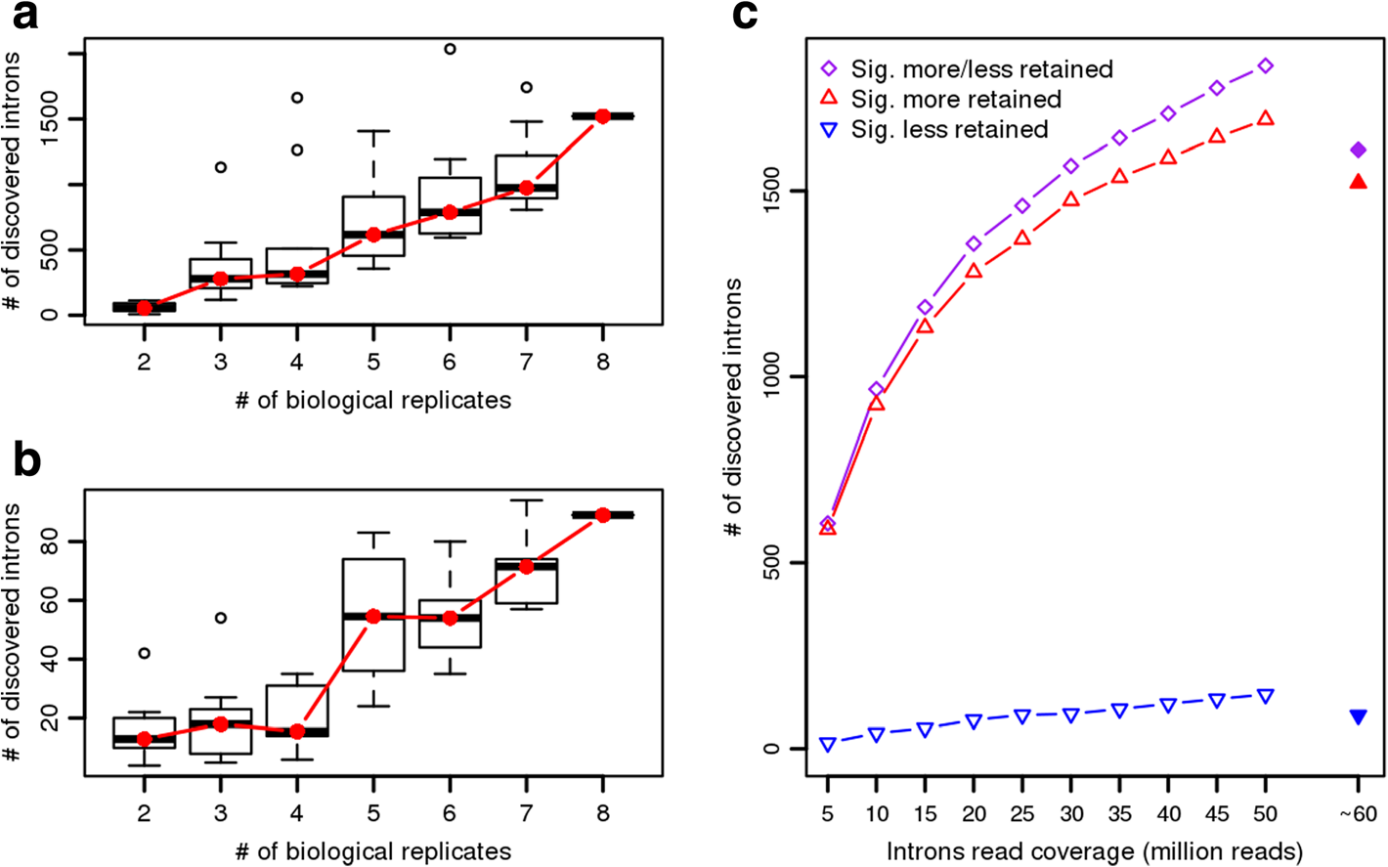
The effect of sample size and sequencing library size sensitivity. **a** The number of significantly higher retained introns in ZRSR2mut samples vs controls, relative to the various number of biological replicates. **b** Similar to the panel A but for the significantly less retained introns in ZRSR2mut samples. **c** The significantly higher retained introns in ZRSR2mut samples vs controls, relative to the number of reads mapped to the introns and exons. A *p*_*adj*_ < 0.01 threshold was used for all analyses. The data points on the far right in each panel (8 biological replicates in panels **a** and **b**; ~60M reads in panel **c** represent the complete MDS dataset used in the analysis. This leads to zero variance in panels **a** and **b** because the resampling size for the complete data is 1 (8 ZRSR2mut vs 8 controls), and less significantly differential IR events compared to the resampled in panel C due to variable size datasets. The conditions of the resampled data in panel C are idealistic, as in these analyses the overall mapped reads for all samples are assumed to be equal (as opposed to in the MDS data where it varies from 51-75 million); hence their number of retained introns are higher compared to the real MDS data

Finally, we studied the effect of the number of biological replications and the intron read coverage levels again using the MDS dataset. To investigate the effect of biological replication, we randomly picked 2 to 8 MDS, ZRSR2mut and control samples for our analysis with IntEREst-DESeq2 (*i.e. DESeq2Interest()* ) and repeated this 10 times. As expected, the results reveal that increasing numbers of biological replicates lead to a discovery of an increased number of statistically significant IR events (Fig. 4a, b). This observation is similar to what has been observed earlier with gene expression analyses [35].

A similar trend was also observed when analyzing the effect of intron/exon read coverage levels. Here we distributed 5-50 million reads according to the relative retention levels of the introns and exon-exon junction levels (based on the complete data) in each sample, followed by analysis with IntEREst-DESeq2. In our analyses we assumed that the quality and read coverage is equal in all the individual MDS datasets. As a result, we observed that an increase in the sequencing library size leads to a discovery of increasing numbers of introns showing statistically significant deviation in the IR levels. However, the slope of increase of the number of discovered IR events decreases and levels off at the highest library sizes (more than 35M; Fig. 4c).

## Conclusion

Here we present IntEREst, an R package for intron retention and exon-exon junction analysis. Our method is able to extract the significantly retained introns and carry out intra- and inter-sample comparisons of the retention levels of the introns and exon junction levels. We used IntEREst to analyze the publicly available MDS data [17] and our results confirm that mutations in the *ZRSR2* gene, a component of the minor spliceosome involved in recognition of 3΄ splice site of the U12-type introns, leads to increased IR particularly with the U12-type introns. Furthermore, our results show that compared to the U2-type introns, the IR of U12-type introns is already higher in the control samples, but the mutations in the *ZRSR2* gene further exacerbate the IR in the patient cells. These conclusion are further supported by our analysis of Maize data with a mutations in plant ortholog of the *ZRSR2* gene, which, similarly to human data, also show strong bias towards increased IR of the U12-type, but not U2-type introns. The introns showing significantly higher or lower IR in the ZRSR2mut samples vs control samples in MDS dataset that we discovered using the IntEREst-DESeq2 (Additional file 2) overlap with the introns identified by IRFinder and IntEREst-edgeR. Furthermore, our results not only detect the same IR events reported in the original study by Madan et al. [17], but we also discovered additional significant IR events featuring both the U12- and U2-type introns.

The resampling analysis of ZRSR2mut vs control samples show that by including more biological replications and considering a larger sequencing library size, increasing number of significant IR events can be discovered. While the maximum number of biological replicates (eight) used in this study is not sufficient to estimate the optimal required for IR discovery, we note that library sizes with more than 35M mapped reads start to approach the point where the improvements in detecting novel IR events are marginal. In sum, we believe that IntEREst is a reliable tool in R/Bioconductor environment for detailed intron retention analysis of RNAseq datasets.

## Availability and requirements

IntEREst is implemented as an R package freely available at the Bioconductor repository.

Project name: IntEREst

Archived version: 1.2.2

Project home page: https://github.com/gacatag/IntEREst/

and https://bioconductor.org/packages/release/bioc/html/IntEREst.html

Operating system(s): Platform independent

Programming language: R

Other requirements: R v 3.4 or higher

License: GPL

Any restrictions to use by non-academics: No restrictions

## Additional files

Additional file 1: HTML file including all the running scripts and supplementary figures and tables. (HTML 789 kb)

Additional file 2: Tab delimited text file that includes the differentially retained introns, when comparing the ZRSR2mut samples to the controls using the IntEREst-DESeq2. (TSV 634 kb)

## Abbreviations

FPKM: Fragments per Kilo base-pair per Million reads
IR: Intron Retention
PSI: Percent Spliced In
