# Supplementary material for: IntEREst: intron-exon retention estimator
Source: BMC Bioinformatics. 2018 Apr 11;19:130. doi: 10.1186/s12859-018-2122-5 (PMC5896110; doi:10.1186/s12859-018-2122-5)
Supplement: Supplementary file 1 — HTML file including all the running scripts and supplementary figures and tables. (HTML 789 kb) [file 12859_2018_2122_MOESM1_ESM.html]

Supplementary 1


# Supplementary 1

#### *Ali Oghabian*

#### *2018-02-09*

Here we present supplementary data together with the scripts that were used to generate the results mentioned in the paper. The document is comprised of the following sections:

- Differential intron retention analysis
  - Mapping read sequences and intron retention analysis
  - Building reference
  - Retention estimation of the introns
  - Discovering introns with strong increased/decreased retention
  - Filtering genes with low intron retention levels
- Performance comparisons
  - Comparing IntEREst-DESeq2 to IntEREst-edgeR
  - Comparing IntEREst-DESeq2 to IntEREst-DEXSeq
  - Comparing IntEREst-DESeq2 results to the retained introns discovered by IRFInder
  - Comparing IntEREst-DESeq2 results to the IRs reported in the MDS study
- Sensitivity analysis
  - Sample size analysis
  - Read coverage analysis
- Differential retention analysis of Maize data

Note that all scripts initiated with a `# Time demanding` comment are time-demanding however, the results of these scripts are available as R data objects in GitHub. Throughout this document, when you are running the other scripts which are not time-consuming, if needed, these results would initially be loaded in R. Also note that running the scripts requires that the working directory in R is set to the path of the local copy of the GitHub repository. The working directory in R can be changed with the `setwd()` command.

# Differential intron retention analysis

We compare the intron retention (IR) variations of U12 type introns to the U2 type (comparing ZRSR2mut samples vs controls), using the DESeq2 based function of IntEREst (IntEREst-DESeq).

## Mapping read sequences and preprocessing

Here, we describe the preprocessing steps, *e.g.* mapping the sequence reads to the genome and running the `interest()` function to measure the number of reads that map to the introns of the genes. The `preprocessing.sh` file in the `scripts` folder (in the GitHub repository) includes scripts that were used to download and map the sequencing reads. It also changes the read names with the intention that the paired reads be recognized as pairs when read in R; **the read IDs of paired reads must be identical**. The `preprocessing.sh` file can be run in Linux environment if `fastq-dump` (from the SRA Toolkit), `Tophat2`, `Bowtie2`, `sed` and `samtools`are installed. If you intend to run `preprocessing.sh`, before running you need to set the working directory to the `scripts` folder and also set the following environmental variables:

- `RAWDATAPATH`: Path to the folder containing all fastq files.
- `MAPPINGPATH`: Path to write and access all mapped sequence reads bam/sam files.
- `indexPATH`: Path to the Bowtie2 index files including the genome name. If the path to the index files is ~/bowtie2/indexes/hg19NoColor/ for hg19 (given that the necessary files are available in this path with hg19.\* filenames) set the indexPath as ~/bowtie2/indexes/hg19NoColor/hg19 .

Note that `Tophat2` and `samtools` were all run with their default parameters. We ran all the analyses on a Linux (CentOS release 7.3.1611) server with 64 AMD Opteron Processors (6274) and a total of ~528 GB memory capacity.

## Building reference

First, a reference data-frame was built; The hg19 and UCSC was used. The exonic regions were collapsed to prevent any biases in the intron retention level (IR) calculation that may be introduced by the read counts of exons overlapping the studied introns. Note that the following analyses are time demanding. You can skip and continue from ‘Discovering introns with strong increase/decrease in retention’.

```
# Time demanding
library(IntEREst)
refseqRef<- referencePrepare (sourceBuild="UCSC", ucscGenome="hg19",
    addCollapsedTranscripts=TRUE, ignore.strand=FALSE)
```

After building the reference, the U12-type introns were annotated. If an intron was not annotated as a U12-type or a U2-type we considered it as U2-type (*i.e.* the parameter setting `setNaAs= "U2"` was used).

```
# Time demanding
library(BSgenome.Hsapiens.UCSC.hg19)
refseqAnnoMat<- annotateU12(
    pwmU12U2= list(
        pwmU12db[[1]][,11:17],
        pwmU12db[[2]],
        pwmU12db[[3]][,38:40],
        pwmU12db[[4]][,11:17],
        pwmU12db[[5]][,38:40]),
    pwmSsIndex= list(
        indexDonU12=1, 
        indexBpU12=1, 
        indexAccU12=3, 
        indexDonU2=1, 
        indexAccU2=3), 
    referenceChr= refseqRef[,"chr"], 
    referenceBegin= as.numeric(refseqRef[,"begin"]), 
    referenceEnd= as.numeric(refseqRef[,"end"]), 
    referenceIntronExon= refseqRef[,"int_ex"],
    intronExon= "intron",
    matchWindowRelativeUpstreamPos= c(NA,-29,NA,NA,NA),
    matchWindowRelativeDownstreamPos= c(NA,-9,NA,NA,NA), 
    minMatchScore= c( rep("80%", 2), "40%", "80%",  "40%"), 
    refGenome= BSgenome.Hsapiens.UCSC.hg19, 
    setNaAs= "U2", 
    annotateU12Subtype= TRUE)
```

## Retention estimation of the introns

To estimate the intron retention (IR) levels the `interest()` function was used. We ran the analysis on 40 computing cores while at each attempt maximum 1 million paired reads were analyzed. For the normalization we scaled the IR levels to the length of the introns and also the number of fragments mapped to the gene.

```
# Time demanding
outDir<-"/data1/ali/develop/sequencing/package/testInterestComplete/new/"
MDS_BAMFILES<- paste(
    "/data1/ali/develop/sequencing/package/testInterestComplete/validation/",
        c(
            "SRR1691633/SRR1691633_ZRSR2Mut.bam", 
            "SRR1691634/SRR1691634_ZRSR2Mut.bam",
            "SRR1691635/SRR1691635_ZRSR2Mut.bam",
            "SRR1691636/SRR1691636_ZRSR2Mut.bam",
            "SRR1691637/SRR1691637_ZRSR2Mut.bam",
            "SRR1691638/SRR1691638_ZRSR2Mut.bam",
            "SRR1691639/SRR1691639_ZRSR2Mut.bam",
            "SRR1691640/SRR1691640_ZRSR2Mut.bam",
            "SRR1691641/SRR1691641_WT.bam",
            "SRR1691642/SRR1691642_WT.bam",
            "SRR1691643/SRR1691643_WT.bam",
            "SRR1691644/SRR1691644_WT.bam",
            "SRR1691645/SRR1691645_Normal.bam",
            "SRR1691646/SRR1691646_Normal.bam",
            "SRR1691647/SRR1691647_Normal.bam",
            "SRR1691648/SRR1691648_Normal.bam"
        ), sep="")
# Set sample names
names(MDS_BAMFILES)<-  gsub(".bam","", gsub(".*/","", MDS_BAMFILES))

for(i in 1:length(MDS_BAMFILES)){
    dir.create(paste(outDir, names(MDS_BAMFILES)[i],
        sep="/"), recursive = TRUE)
   interest(
        bamFileYieldSize=1000000,
        bamFile=MDS_BAMFILES[i],
        isPaired=TRUE,
        isPairedDuplicate=FALSE,
        isSingleReadDuplicate=NA,
        reference=refseqRef,
        referenceGeneNames=refseqRef[,"collapsed_transcripts_id"],
        referenceIntronExon=refseqRef[,"int_ex"],
        repeatsTableToFilter= c(),
        junctionReadsOnly=FALSE,
        outFile=paste(outDir, names(MDS_BAMFILES)[i], 
            "interestRes.tsv", sep="/"),
        logFile=paste(outDir, names(MDS_BAMFILES)[i], 
            "log.txt", sep="/"),
        method=c"IntRet",
        clusterNo=40,
        returnObj=FALSE, 
        scaleLength= TRUE, 
        scaleFragment= TRUE
    )
}
```

The running time for each sample (with 40 computing cores in use) was as follows:

| Sample name | Running time (Secs) |
| --- | --- |
| SRR1691633\_ZRSR2Mut | 11763.74 |
| SRR1691634\_ZRSR2Mut | 11453.52 |
| SRR1691635\_ZRSR2Mut | 11305.33 |
| SRR1691636\_ZRSR2Mut | 11675.77 |
| SRR1691637\_ZRSR2Mut | 11630.58 |
| SRR1691638\_ZRSR2Mut | 11282.91 |
| SRR1691639\_ZRSR2Mut | 12713.78 |
| SRR1691640\_ZRSR2Mut | 11502.06 |
| SRR1691641\_WT | 14215.48 |
| SRR1691642\_WT | 12938.56 |
| SRR1691643\_WT | 11699.02 |
| SRR1691644\_WT | 12169.6 |
| SRR1691645\_Normal | 11697.54 |
| SRR1691646\_Normal | 11427.7 |
| SRR1691647\_Normal | 11622.74 |
| SRR1691648\_Normal | 11934.94 |

**Table S1.** Running time of `interest()` function for each MDS sample (Number of computing cores = 40).

After the IR of all samples were estimated, we made an object of class SummarizedExperiment that contains the IR results of all samples, and the intron/exon and sample annotations.

```
# Read results and build SummarizedExperiment object
mdsRefObj<-readInterestResults(
    resultFiles=paste(outDir, names(MDS_BAMFILES), 
            "interestRes.tsv", sep="/"), 
    sampleNames=names(MDS_BAMFILES), 
    sampleAnnotation=data.frame( 
        type=c(rep("ZRSR2mut",8), rep("ZRSR2wt",4), rep("HEALTHY",4)),
        test_ctrl=c(rep("test",8), rep("ctrl",8))), 
    commonColumns=1:9, freqCol=10, scaledRetentionCol=11,
    scaleLength=TRUE, scaleFragment=TRUE, reScale=TRUE, 
    geneIdCol="collapsed_transcripts_id")

# update the object with the intron type (U12- or U2-type) annotations
mdsRefObj<- updateRowDataCol(mdsRefObj,  "intron_type", refseqAnnoMat[,1])

save(mdsRefObj, file="./mdsRefObj.rda")
```

The `mdsRefObj` object has been included in the GitHub repository. In the next section, we will load and use it to make plots and perform downstream analyses.

## Discovering introns with strong increase/decrease in retention

We extract introns that featured strong differential IRs when comparing ZRSR2mut to the controls, using the DESeq2 based function of IntEREst (IntEREst-DESeq2). To carry out this analysis we extract the introns which their retentions increase/decrease significantly compared to the decrease/ increase of the junction levels of their flanking exons. We initially, build a reference data.frame from UCSC without collapsing the exons. Next, we keep a single copy of each set of repeating exons. We run the IntEREst in the exon-exon junction mode. and finally we merge the exon junction results to the previously used intron retention results so that the resulting object would include the retention level of the introns together with the mean of the junction levels of their flanking exons. Finally we perform DESeq2 differential test to get the relevant p values. The following runs are however time demanding; You can skip this step and continue with the remaining analysis.

```
# Time demanding
library(BSgenome.Hsapiens.UCSC.hg19)
refseqUncollapsed<- referencePrepare (sourceBuild="UCSC", ucscGenome="hg19",
    addCollapsedTranscripts=FALSE, collapseExons=FALSE, ignore.strand=FALSE)

# Union exons of transcripts with overlapping exons
# Keep one copy from each repeating set of exons, only
refExDf<- unionRefTr(referenceChr= refseqUncollapsed[,"chr"], 
    referenceBegin= as.numeric(refseqUncollapsed[,"begin"]), 
    referenceEnd= as.numeric(refseqUncollapsed[,"end"]), 
    referenceTr=as.character(refseqUncollapsed[,"transcript_id"]),
    referenceIntronExon=refseqUncollapsed[,"int_ex"],
    intronExon="exon",
    silent=FALSE)
save(refExDf,file="./refExDf.rda")

outDir="/data1/ali/develop/sequencing/package/testInterestComplete/newEx/"
setwd(outDir)
MDS_BAMFILES<- paste(
    "/data1/ali/develop/sequencing/package/testInterestComplete/validation/",
        c(
            "SRR1691633/SRR1691633_ZRSR2Mut.bam", 
            "SRR1691634/SRR1691634_ZRSR2Mut.bam",
            "SRR1691635/SRR1691635_ZRSR2Mut.bam",
            "SRR1691636/SRR1691636_ZRSR2Mut.bam",
            "SRR1691637/SRR1691637_ZRSR2Mut.bam",
            "SRR1691638/SRR1691638_ZRSR2Mut.bam",
            "SRR1691639/SRR1691639_ZRSR2Mut.bam",
            "SRR1691640/SRR1691640_ZRSR2Mut.bam",
            "SRR1691641/SRR1691641_WT.bam",
            "SRR1691642/SRR1691642_WT.bam",
            "SRR1691643/SRR1691643_WT.bam",
            "SRR1691644/SRR1691644_WT.bam",
            "SRR1691645/SRR1691645_Normal.bam",
            "SRR1691646/SRR1691646_Normal.bam",
            "SRR1691647/SRR1691647_Normal.bam",
            "SRR1691648/SRR1691648_Normal.bam"
        ), sep="")

# Set sample names

names(MDS_BAMFILES)<-  gsub(".bam","", gsub(".*/","", MDS_BAMFILES))

for(i in 1:length(MDS_BAMFILES)){
    dir.create(paste(outDir, names(MDS_BAMFILES)[i],
        sep="/"), recursive = TRUE)
   interest(
        bamFileYieldSize=1000000,
        bamFile=MDS_BAMFILES[i],
        isPaired=TRUE,
        isPairedDuplicate=FALSE,
        isSingleReadDuplicate=NA,
        reference=refExDf,
        referenceGeneNames=refExDf[,"transcripts_id"],
        referenceIntronExon=refExDf[,"int_ex"],
        repeatsTableToFilter= c(),
        junctionReadsOnly=TRUE,
        outFile=paste(outDir, names(MDS_BAMFILES)[i], 
            "interestRes.tsv", sep="/"),
        logFile=paste(outDir, names(MDS_BAMFILES)[i], 
            "log.txt", sep="/"),
        method=c("ExEx"),
        clusterNo=20,
        returnObj=FALSE, 
        scaleLength= FALSE, 
        scaleFragment= TRUE,
        bpparam= SnowParam(workers=25)
    )
}

mdsExRefObj<-readInterestResults(
    resultFiles=paste(outDir, names(MDS_BAMFILES), 
            "interestRes.tsv", sep="/"), 
    sampleNames=names(MDS_BAMFILES), 
    sampleAnnotation=data.frame( 
        type=c(rep("ZRSR2mut",8), rep("ZRSR2wt",4), rep("HEALTHY",4)),
        test_ctrl=c(rep("test",8), rep("ctrl",8))), 
    commonColumns=1:5, freqCol=6, scaledRetentionCol=7,
    scaleLength=FALSE, scaleFragment=TRUE, reScale=FALSE, 
    geneIdCol="transcripts_id")

save(mdsExRefObj, 
    file="./mdsExRefObj.rda")

load("./mdsRefObj.rda")
load("./mdsExRefObj.rda")
mdsRfObjIntBool<-rowData(mdsRefObj)$int_ex=="intron"
mdsRefObjInt<- mdsRefObj[mdsRfObjIntBool,]
mdsRefIntExObj<- interestResultIntEx(intObj=mdsRefObjInt, exObj=mdsExRefObj, 
    mean.na.rm=TRUE, postExName="ex_junc" )
save(mdsRefIntExObj,file="./mdsRefIntExObj.rda")
ddsDiff<- deseqInterest(mdsRefIntExObj,  
    design=~test_ctrl+test_ctrl:intronExon, 
    sizeFactor=rep(1,nrow(colData(mdsRefIntExObj))), 
    contrast=list("test_ctrltest.intronExonintron",
        "test_ctrlctrl.intronExonintron"),
    parallel=TRUE,
    BPPARAM = SnowParam(workers=10))
save(ddsDiff, file="./ddsDiff.rda")

# Set adjusted p value threshold
pThresh<- 0.01
#get count data
cntMdsRefIntExObj<-counts(mdsRefIntExObj)
#Change colnames for more clarity
colnames(cntMdsRefIntExObj)[1:16]<- 
    paste(colnames(cntMdsRefIntExObj)[1:16], "IR", sep="_")
sigMoreOrLess<- rep(NA, nrow(ddsDiff))
sigMoreOrLess[which(ddsDiff$padj<pThresh & ddsDiff$log2FoldChange>0)]<- "more"
sigMoreOrLess[which(ddsDiff$padj<pThresh & ddsDiff$log2FoldChange<0)]<- "less"

# Annotate gene names of the collpased transcripts
library (RMySQL)
con <- dbConnect(
    RMySQL::MySQL(), 
    host="genome-mysql.soe.ucsc.edu",
    user="genome",
    dbname="hg19")

genAnnoTabl<- dbGetQuery(
    con, 
    "SELECT name,geneSymbol FROM knownGene,kgXref  where kgID = name")
genAnno<- genAnnoTabl[,2]
names(genAnno)<- genAnnoTabl[,1]

# Annotate gene names of introns

library(GenomicRanges)
refseqUncollapsedInt<- 
    refseqUncollapsed[which(refseqUncollapsed$int_ex=="intron"),]

geneSymb<- rep(NA, nrow(rowData(mdsRefIntExObj)))
refseqUncollapsedIntGr<-  GRanges(refseqUncollapsedInt$chr,
    IRanges(refseqUncollapsedInt$begin, 
        refseqUncollapsedInt$end))
mdsRefIntExObjGr<-  GRanges(rowData(mdsRefIntExObj)$chr,
    IRanges(rowData(mdsRefIntExObj)$begin, 
        rowData(mdsRefIntExObj)$end))

intronsMatch<- suppressWarnings(
    findOverlaps(mdsRefIntExObjGr, refseqUncollapsedIntGr, type="equal") )
tmpApply<- tapply(
    subjectHits(intronsMatch), 
    queryHits(intronsMatch), 
    function(tmp)
        paste(sort(unique(genAnno[refseqUncollapsedInt[tmp,"transcript_id"]]),
            decreasing=FALSE), collapse=","))
geneSymb[as.numeric(names(tmpApply))]<- as.vector(tmpApply)

mdsRefIntExObjGenAnno<- sapply(
    strsplit(rowData(mdsRefIntExObj)[,"collapsed_transcripts"], split=","),
    function(tmp) 
        paste(sort(unique(genAnno[tmp]), decreasing=FALSE), collapse=","))
# Add delts psi values
psiMds<-psi(x=mdsRefIntExObj, 
    intCol= which(colData(mdsRefIntExObj)$intronExon=="intron"), 
    exCol=which(colData(mdsRefIntExObj)$intronExon=="exon"))

psiMdsTest<- psiMds[,which(colData(mdsRefIntExObj)$test_ctrl[
        colData(mdsRefIntExObj)$intronExon=="intron"]=="test")]

psiMdsCtrl<- psiMds[,which(colData(mdsRefIntExObj)$test_ctrl[
        colData(mdsRefIntExObj)$intronExon=="intron"]=="ctrl")]

difPsiAll<- (rowMeans(psiMdsTest)-rowMeans(psiMdsCtrl))


# Write differential IR data
write.table(
    data.frame(
        as.data.frame(rowData(mdsRefIntExObj))[,1:6],
        gene_symbol= geneSymb,
        as.data.frame(rowData(mdsRefIntExObj))[,7:8],
        collapsed_gene_symbol= mdsRefIntExObjGenAnno,
        as.data.frame(rowData(mdsRefIntExObj))[,9:10],
        as.data.frame(cntMdsRefIntExObj),
        as.data.frame(ddsDiff),
        sig_more_or_less=sigMoreOrLess,
        delta_psi=difPsiAll )[which(!is.na(sigMoreOrLess)),], 
    file="./differential-IR.tsv",
    col.names=TRUE,
    row.names=FALSE,
    quote=FALSE, 
    sep='\t'
    )
```

The following scripts load the required data (generated by the previous scripts) and checks its contents:

```
pThresh<- 0.01
load(file="./mdsRefIntExObj.rda")
load(file="./ddsDiff.rda")
# index of significantly more retained introns
indUpMdsRefDdsObjInt<- which(ddsDiff$padj<pThresh & 
    ddsDiff$log2FoldChange>0)
# index of significantly less retained introns
indDnMdsRefDdsObjInt<- which(ddsDiff$padj<pThresh & 
    ddsDiff$log2FoldChange<0)
# Type of significantly retained introns
table(rowData(mdsRefIntExObj)[indUpMdsRefDdsObjInt, "intron_type"])
```

```
## 
##    U12 U12/U2     U2 
##    269      4   1248
```

```
# Type of significantly less retained introns
table(rowData(mdsRefIntExObj)[indDnMdsRefDdsObjInt, "intron_type"])
```

```
## 
##    U12 U12/U2     U2 
##      0      0     89
```

The following scripts initially loads the results of the previous scripts and runs a statistical test to see whether the log FC of IR levels of U12 type introns are higher than that of U2 type introns. Next suitable colours and shapes of plot points (pch) are defined which will be used in the plot to distinguish significantly more/less retained U12 type introns from the U2 type, and from the introns that have not significantly varried across the studied samples.

```
load("./ddsDiff.rda")
load("./mdsRefIntExObj.rda")
pThresh<- 0.01

mdsRefIntObj<- mdsRefIntExObj[,colData(mdsRefIntExObj)$intronExon=="intron"]
lfcRes<- lfc(mdsRefIntObj, fcType= "edgeR", 
    sampleAnnoCol="test_ctrl",sampleAnnotation=c("ctrl","test"))
```

```
## Design matrix not provided. Switch to the classic mode.
```

```
# Build index for U2 introns
u2TF<- rep(FALSE,length(lfcRes))
u2TF[ which(rowData(mdsRefIntObj)[, "intron_type"]=="U2" & 
    rowData(mdsRefIntObj)[, "int_ex"]=="intron")]<- TRUE
u2TF[ which(rowData(mdsRefIntObj)[, "intron_type"]=="U12/U2" & 
    rowData(mdsRefIntObj)[, "int_ex"]=="intron")]<- FALSE
u2TF[u12Index(mdsRefIntObj, intExCol="int_ex", 
    intTypeCol="intron_type")]<- FALSE
#Check number of U2 introns
length(which(u2TF))
```

```
## [1] 228014
```

```
# Check if the FC retention increase is significant
lfcVec<- c(lfcRes[u2TF], lfcRes[u12Index(mdsRefIntObj, intExCol="int_ex",
    intTypeCol="intron_type")])
ord<- c(rep(1, length(lfcRes[u2TF])),
    rep(2, length(lfcRes[u12Index(mdsRefIntObj, intExCol="int_ex",
        intTypeCol="intron_type")])))
table(ord)
```

```
## ord
##      1      2 
## 228014    510
```

```
library(clinfun)
jtRes<- jonckheere.test(lfcVec, ord, alternative = "increasing",
    nperm=1000)
jtRes
```

```
## 
##  Jonckheere-Terpstra test
## 
## data:  
## JT = 98984000, p-value = 0.001
## alternative hypothesis: increasing
```

```
# Set proper colours and point symbols (pch) for plotting to distinguish 
# U12-type more/less retained introns from the U2-type more/less retained
cols<- rep("lightgrey", nrow(rowData(mdsRefIntExObj)))
cols[rowData(mdsRefIntExObj)[, "intron_type"]=="U12"]<- "black"
cols[rowData(mdsRefIntExObj)[, "intron_type"]=="U12" &  
    ddsDiff$padj<pThresh ]<- "red"
pchs=rep(21, nrow(rowData(mdsRefIntExObj)))
pchs[ddsDiff$padj<pThresh &  
    ddsDiff$log2FoldChange>0]<- 24
pchs[ddsDiff$padj<pThresh &  
    ddsDiff$log2FoldChange<0]<- 25


#Number of significantly more retained U12 introns
(u12UpNo<-length(which(pchs==24 & cols=="red")))
```

```
## [1] 269
```

```
#Number of significantly lower retained U12 introns
(u12DnNo<-length(which(pchs==25 & cols=="red")))
```

```
## [1] 0
```

```
#Number of U12 type introns
(u12No<- length(which(rowData(mdsRefIntExObj)[, "intron_type"]=="U12")))
```

```
## [1] 510
```

```
#Number of U12 or U2 type introns
(u12u2No<- length(which(rowData(mdsRefIntExObj)[, "intron_type"]=="U12" |
    rowData(mdsRefIntExObj)[, "intron_type"]=="U2")))
```

```
## [1] 228524
```

```
#Percentage of significantly more retained U12 introns
100*u12UpNo/u12No
```

```
## [1] 52.7451
```

```
#Percentage of significantly less retained U12 introns
100*u12DnNo/u12No
```

```
## [1] 0
```

```
#Number of Significantly more retained introns (U12 and U2)
length(which(pchs==24))
```

```
## [1] 1521
```

```
#Number of Significantly less retained introns (U12 and U2)
length(which(pchs==25))
```

```
## [1] 89
```

```
#Number of significantly more retained U2 introns
(u2UpNo<-length(which(pchs==24 & cols=="lightgrey")))
```

```
## [1] 1252
```

```
#Number of significantly less retained U2 introns
(u2DnNo<-length(which(pchs==25 & cols=="lightgrey")))
```

```
## [1] 89
```

```
#Number of U2 type introns
(u2No<-length(which(rowData(mdsRefIntExObj)[, "intron_type"]=="U2")))
```

```
## [1] 228014
```

```
#Percentage of significantly more retained U2 introns
100*u2UpNo/u2No
```

```
## [1] 0.5490891
```

```
#Percentage of significantly less retained U2 introns
100*u2DnNo/u2No
```

```
## [1] 0.03903269
```

The following scripts, merge the information shown in Fig.1 A and B and illustrate all in a single plot.

```
ddsDiffBm<- log(ddsDiff$baseMean, base=10)
par(mar=c(4,4,2,1))
plot(ddsDiffBm, ddsDiff$log2FoldChange, 
    col=0, pch=16, xlab="", ylab=expression("fc (log"[2]*")"), mgp=c(2.9,1,0))
points(ddsDiffBm[cols=="lightgrey"&pchs==21], 
    ddsDiff$log2FoldChange[cols=="lightgrey"&pchs==21], 
    col="lightgrey", pch=21)
points(ddsDiffBm[cols=="lightgrey"&pchs==24], 
    ddsDiff$log2FoldChange[cols=="lightgrey"&pchs==24], 
    col="darkgrey", pch=24)
points(ddsDiffBm[cols=="lightgrey"&pchs==25], 
    ddsDiff$log2FoldChange[cols=="lightgrey"&pchs==25], 
    col="darkgrey", pch=25)
points(ddsDiffBm[cols=="black"&pchs==21], 
    ddsDiff$log2FoldChange[cols=="black"&pchs==21], col="black",
        pch=21)
points(ddsDiffBm[cols=="red"&pchs==24], 
    ddsDiff$log2FoldChange[cols=="red"&pchs==24], 
        col="red", pch=24)
points(ddsDiffBm[cols=="red"&pchs==25], 
    ddsDiff$log2FoldChange[cols=="red"&pchs==25], 
        col="red", pch=25)
 mtext(expression("cpm (log"[2]*")"), side=1, outer=F, line=2, cex=.7)
legend("bottomright", 
    legend=c("U2 NOT changed", "U2 significantly retained", 
        "U2 significantly LESS retained", "U12 NOT changed", 
        "U12 significantly retained", "U12 significantly LESS retained"), 
    col=c("lightgrey", "darkgrey", "darkgrey", "black", "red", "red"), 
    pch=c(21, 24, 25, 21, 24, 25), cex=.7 )
```

**Fig. S1.** The plot shows IR fold change (ZRSR2mut vs control) vs normalized retention levels of U12- and U2-type introns in the filtered data. Significantly higher and lower retained U12 and U2-type introns have been distinguished from the unchanged introns.

The following scripts generate the figures Fig. 1 and 2 in the paper.

```
library(grid)

# function to draw curly braces in red
# x1...y2 are the ends of the brace
# for upside down braces, x1 > x2 and y1 > y2
CurlyBraces <- function(x0, x1, y0, y1, pos = 1, direction = 1, depth = 1) {

    a=c(1,2,3,48,50)    # set flexion point for spline
    b=c(0,.2,.28,.7,.8) # set depth for spline flexion point

    curve = spline(a, b, n = 50, method = "natural")$y * depth

    curve = c(curve,rev(curve))

    if (pos == 1){
        a_sequence = seq(x0,x1,length=100)
        b_sequence = seq(y0,y1,length=100)  
    }
    if (pos == 2){
        b_sequence = seq(x0,x1,length=100)
        a_sequence = seq(y0,y1,length=100)      
    }

    # direction
    if(direction==1)
        a_sequence = a_sequence+curve
    if(direction==2)
        a_sequence = a_sequence-curve

    # pos
    if(pos==1)
        lines(a_sequence,b_sequence, lwd=1.5,   xpd=NA) # vertical
    if(pos==2)
        lines(b_sequence,a_sequence, lwd=1.5, xpd=NA) # horizontal

}

#Plotting

load("./ddsDiff.rda")
pThresh<- 0.01
cols<- rep("lightgrey", nrow(rowData(mdsRefIntExObj)))
cols[rowData(mdsRefIntExObj)[, "intron_type"]=="U12"]<- "black"
cols[rowData(mdsRefIntExObj)[, "intron_type"]=="U12" &  
    ddsDiff$padj<pThresh ]<- "red"
pchs=rep(21, nrow(rowData(mdsRefIntExObj)))
pchs[ddsDiff$padj<pThresh &  
    ddsDiff$log2FoldChange>0]<- 24
pchs[ddsDiff$padj<pThresh &  
    ddsDiff$log2FoldChange<0]<- 25

# Output figure
tiff("./figures/fig1.tif", width=1200, height=1200, res=300, pointsize=5)
par(lwd=1)
par(cex=1.1)
par(cex.axis=1.1)
par(mfrow=c(2,1))
par(mar=c(5,4.5,2,1))
logddsDiff<- log(ddsDiff$baseMean, base=10)
par(mar=c(5,4.5,2,1))
plot(logddsDiff, 
    ddsDiff$log2FoldChange, col=0, pch=16, xlab="", cex.lab=1.1, 
    ylab=expression("fc (log"[2]*")"), mgp=c(2.9,1,0))
points(logddsDiff[cols=="black"&pchs==21], 
    ddsDiff$log2FoldChange[cols=="black"&pchs==21], col="black", 
        pch=21)
points(logddsDiff[cols=="red"&pchs==24], 
    ddsDiff$log2FoldChange[cols=="red"&pchs==24], col="green", 
    pch=24)
points(logddsDiff[cols=="red"&pchs==25], 
    ddsDiff$log2FoldChange[cols=="red"&pchs==25], col="red", 
    pch=25)
mtext(expression("normalized retention levels (log"[10]*")"), side=1, 
    outer=FALSE, line=2.5, cex=1.1)
legend("bottomright", legend=c("U12 NOT changed", "U12 significantly retained",
    "U12 significantly LESS retained"), col=c("black", "green", "red"), 
    pch=c(21, 24, 25), cex=1.1)
title(main = "(A)")

plot(logddsDiff, ddsDiff$log2FoldChange, cex.lab=1.1, 
    col=0, pch=16, xlab="", ylab=expression("fc (log"[2]*")"), mgp=c(2.9,1,0))
points(logddsDiff[cols=="lightgrey"&pchs==21], 
    ddsDiff$log2FoldChange[cols=="lightgrey"&pchs==21], 
        col="black", pch=21)
points(logddsDiff[cols=="lightgrey"&pchs==24], 
    ddsDiff$log2FoldChange[cols=="lightgrey"&pchs==24], col="green", 
        pch=24)
points(logddsDiff[cols=="lightgrey"&pchs==25],
    ddsDiff$log2FoldChange[cols=="lightgrey"&pchs==25], 
        col="red", pch=25)
mtext(expression("normalized retention levels (log"[10]*")"), side=1, outer=F, 
    line=2.5, cex=1.1)
legend("bottomright", legend=c("U2 NOT changed", "U2 significantly retained",
    "U2 significantly LESS retained"), col=c( "black", "green", "red"), 
    pch=c(21, 24, 25), cex=1.1 )
title(main = "(B)")

dev.off()


# filter transcripts with low number of reads mapped to all its introns

rlCntMdsRefObjInt<- 
    rowMeans(rlog(as.matrix(counts(mdsRefIntExObj))[, 
        colData(mdsRefIntExObj)$intronExon=="intron"]))
mdsMaxRetTr<- tapply(rlCntMdsRefObjInt, 
    as.character(rowData(mdsRefIntExObj)[,"collapsed_transcripts_id"]), 
    max)
inMdsTr<- names(mdsMaxRetTr)[which(as.numeric(mdsMaxRetTr)>1)]
mdsRowFilBool<- 
    rowData(mdsRefIntExObj)[,"collapsed_transcripts_id"] %in% inMdsTr
mdsRefIntExFilRowObj<- mdsRefIntExObj[mdsRowFilBool,]
save(mdsRefIntExFilRowObj,file="./mdsRefIntExFilRowObj.rda")
 
tiff("./figures/fig2.tif", width=1200, height=2400, res=300, pointsize=6)
par(lwd=1)
par(cex=1.5)
par(cex.axis=1.5)
par(cex.lab=1.5)
par(mfrow=c(4,1))
par(mar=c(5,5,2,1))
u12BoxplotNb(mdsRefIntExFilRowObj[,1:16], sampleAnnoCol="type", lasNames=1,
    intExCol="int_ex", intTypeCol="intron_type", intronExon="intron", 
    boxplotNames=c(), outline=FALSE, plotLegend=TRUE, 
    geneIdCol="collapsed_transcripts_id", xLegend="topleft", 
    col=c("pink", "lightblue", "lightyellow"), ylim=c(0,700000), 
    ylab="FPKM", cex.axis=1.5, cex.lab=1.5, cexLegend=1.5, addGrid=TRUE,
    xaxt='n')
axis(1, at=c(8.5, 25.5, 42.5), 
    labels=c("Upstream U2-type intron", "U12-type intron", 
        "Downstream U2-type intron"))
title(main = "(A)", cex.main=1.5)
par(mar=c(6.5,5,2,1))
u12Boxplot(mdsRefIntExFilRowObj[,1:16], sampleAnnoCol="type", 
    intExCol="int_ex",  intTypeCol="intron_type", 
    col=rep(c("orange", "yellow"),3) ,  lasNames=1, 
    outline=FALSE, ylab="FPKM", cex.axis=1.5, intronExon="intron", 
    addGrid=TRUE, boxplotNames= rep(c("U12","U2"), 3))
c(rep("ZRSR2mut",2), 
        rep("ZRSR2wt",2), rep("HEALTHY", 2))
CurlyBraces(.7,2.3,-90000,-90000, pos = 2, direction = 2, depth=20000)
CurlyBraces(3.7,5.3,-90000,-90000, pos = 2, direction = 2, depth=20000)
CurlyBraces(6.7,8.3,-90000,-90000, pos = 2, direction = 2, depth=20000)
mtext(c("ZRSR2mut", "ZRSR2wt", "HEALTHY"), at=c(1.5, 4.5, 7.5), side=1, 
    line=4.2, cex=1.1)
title(main = "(B)", cex.main=1.5)
par(mar=c(5,5,2,1))
u12DensityPlotIntron(mdsRefIntExFilRowObj[,1:16], 
    type= c("U12", "U2Up", "U2Dn", "U2UpDn", "U2Rand"), 
    fcType= "edgeR", sampleAnnoCol="test_ctrl", 
    sampleAnnotation=c("ctrl","test"), intExCol="int_ex", 
    intTypeCol="intron_type", strandCol= "strand", 
    geneIdCol= "collapsed_transcripts_id", naUnstrand=FALSE, col=c(2,3,4,5,6),
    lty=c(1,2,3,4,5), lwd=1, plotLegend=TRUE, cexLegend=1.5, 
    xLegend="topright", yLegend=NULL, 
    legend= c("U12-type intron (n=464)", 
        "U2-type upstream introns (n=266)",
        "U2-type downstream introns (n=464)",
        "U2-type up/down stream introns (n=730)",
        "U2-type random introns (n=464)"), 
    randomSeed=10,
    ylim=c(0,1), xlab=expression("log"[2]*" fold change FPKM"))
title(main = "(C)", cex.main=1.5)
# psi plot
psiMds<-psi(x=mdsRefIntExFilRowObj, 
    intCol= which(colData(mdsRefIntExFilRowObj)$intronExon=="intron"), 
    exCol=which(colData(mdsRefIntExFilRowObj)$intronExon=="exon"))
psiMdsTest<- psiMds[,which(colData(mdsRefIntExFilRowObj)$test_ctrl[
        colData(mdsRefIntExFilRowObj)$intronExon=="intron"]=="test")]
psiMdsCtrl<- psiMds[,which(colData(mdsRefIntExFilRowObj)$test_ctrl[
        colData(mdsRefIntExFilRowObj)$intronExon=="intron"]=="ctrl")]
difPsiAll<- (rowMeans(psiMdsTest)-rowMeans(psiMdsCtrl))
u12Ind<- u12Index(mdsRefIntExFilRowObj, intExCol="int_ex", 
    intTypeCol="intron_type")
plot(density(difPsiAll[-u12Ind], na.rm=TRUE), type='l', lwd=1, lty=2, main="",
    xlab="ΔΨ")
points(density(difPsiAll[u12Ind], na.rm=TRUE), type='l', lwd=1, lty=1, 
    col="red")
text(x=0.05, y = 20, labels="U2", col="black")
text(x=0.4, y = 3, labels="U12", col="red")
title(main = "(D)", cex.main=1.5)
dev.off()
```

## Filtering genes with low intron retention levels

Here we plot density (the frequency distribution) of the log foldchange of retention levels of various types of introns when comparing ZRSR2 mutated samples to the ctrl samples. It shows the density plots of the full MDS data (Fig. S2A) and the filtered data (Fig. S2B) that excludes genes which all of their introns feature low (i.e. <=1) normalized retention. The filtered data features less U12 and U2 type introns with log fold change IRs of ~0. These excluded introns featured low log fold change IRs due to the low read coverage (or possibly low expression of their transcripts).

```
library(IntEREst)
load(file="./mdsRefIntExObj.rda")
load(file="./mdsRefIntExFilRowObj.rda")
par(mfrow=c(2,1))
par(mar=c(5,4,2,1))
u12DensityPlotIntron(
    mdsRefIntExObj[,colData(mdsRefIntExObj)$intronExon=="intron"], 
    type= c("U12", "U2Up", "U2Dn", "U2UpDn", "U2Rand"), 
    fcType= "edgeR", sampleAnnoCol="test_ctrl", 
    sampleAnnotation=c("ctrl","test"), intExCol="int_ex", 
    intTypeCol="intron_type", strandCol= "strand", 
    geneIdCol= "collapsed_transcripts_id", naUnstrand=FALSE, col=c(2,3,4,5,6),
    lty=c(1,2,3,4,5), lwd=1, plotLegend=TRUE, cexLegend=0.7, 
    xLegend="topright", yLegend=NULL, legend=c(), randomSeed=10,
    ylim=c(0,1), xlab=expression("log"[2]*" fold change FPKM"), 
    ylab="Density for the complete data")
```

```
## Design matrix not provided. Switch to the classic mode.
```

```
title(main = "(A)")
par(mar=c(5,4,2,1))
u12DensityPlotIntron(
    mdsRefIntExFilRowObj[,colData(mdsRefIntExObj)$intronExon=="intron"],
    type= c("U12", "U2Up", "U2Dn", "U2UpDn", "U2Rand"), 
    fcType= "edgeR", sampleAnnoCol="test_ctrl", 
    sampleAnnotation=c("ctrl","test"), intExCol="int_ex", 
    intTypeCol="intron_type", strandCol= "strand", 
    geneIdCol= "collapsed_transcripts_id", naUnstrand=FALSE, col=c(2,3,4,5,6),
    lty=c(1,2,3,4,5), lwd=1, plotLegend=TRUE, cexLegend=0.7, 
    xLegend="topright", yLegend=NULL, legend=c(), randomSeed=10,
    ylim=c(0,1), xlab=expression("log"[2]*" fold change FPKM"), 
    ylab="Density for filtered data")
```

```
## Design matrix not provided. Switch to the classic mode.
```

```
title(main = "(B)")
```

**Fig. S2.** Line plots showing the frequency (density) of the fold change of the FPKM normalized IR levels of U12-type introns when comparing ZRSR2mut vs controls for (A) the filtered data that lacks genes that all of its introns are low retained and (B) the complete data. As a control, density plots randomly selected U2-type introns and U2-type introns located in the U12-type intron-containing genes, either up- or downstream of the U12-type introns, are also included.

# Performance comparisons

Here we compare the results obtained by IntEREst-DESeq2, i.e. to those extracted from other methods.

## Comparing IntEREst-DESeq2 to IntEREst-edgeR

Here we compare the log foldchange and p-value results exracted from DESeq2 function of IntEREst to those resulted by the edgeR based function of IntEREst.

```
#Change this path if you prefer the temp files be copied elsewhere that the 
# working directory
tmpPath<- "./"
library(DESeq2)
load(file="./ddsDiff.rda")
glmIntExRef<- glmInterest(mdsRefIntExObj,
    sampleAnnoCol="test_ctrl", sampleAnnotation=c("ctrl","test"),
    geneIdCol= "collapsed_transcripts_id", silent=TRUE, disp="common", 
    design=model.matrix(~test_ctrl+test_ctrl:intronExon, 
    data= colData(mdsRefIntExObj)),
    contrast=c(0,0,-1,1)
    )
save(glmIntExRef, file=paste(tmpPath, "glmIntExRef.rda", sep="/"))
padGlm<- p.adjust(glmIntExRef$table$PValue, method="BH")

length(padGlm)
```

```
## [1] 228535
```

```
pThresh<- 0.01
col1<-"green"
col2<-"lightblue"
col3<-"yellow2"
cols<- rep("black", length(ddsDiff$padj))
cols[which(ddsDiff$padj<pThresh&padGlm<pThresh)]<- col1
cols[which(ddsDiff$padj<pThresh&padGlm>=pThresh)]<- col2
cols[which(ddsDiff$padj>=pThresh&padGlm<pThresh)]<- col3
par(mfrow=c(2,1))
plot(ddsDiff$log2FoldChange, -1*log(ddsDiff$padj, base=10), 
    col=0, pch=16, xlab="FC (log2)", ylab="DESeq2 p-value (-log10)")
points(ddsDiff$log2FoldChange[cols=="black"], 
    -1*log(ddsDiff$padj[cols=="black"], base=10), col=cols[cols=="black"], 
    pch=16)
points(ddsDiff$log2FoldChange[cols==col1], -1*log(ddsDiff$padj[cols==col1],
    base=10), col=cols[cols==col1], pch=16)
points(ddsDiff$log2FoldChange[cols==col2], -1*log(ddsDiff$padj[cols==col2],
    base=10), col=cols[cols==col2], pch=16)
points(ddsDiff$log2FoldChange[cols==col3], -1*log(ddsDiff$padj[cols==col3],
    base=10), col=cols[cols==col3], pch=16)
abline(h=2, col="red", lwd=2)
legend("topleft", legend=c("DESeq2 and GLM p-values < 0.01", 
    "Only DESeq2 p-value < 0.01", "Only GLM p-value < 0.01"), 
    fil=c(col1, col2, col3))
text(-8.5,0.8,"Y = 2",col="red", cex=1.5)
title(main = "(A)")

cols<- rep("black", length(ddsDiff$padj))
cols[which(ddsDiff$padj<pThresh&padGlm<pThresh)]<- col1
cols[which(ddsDiff$padj<pThresh&padGlm>=pThresh)]<- col2
cols[which(ddsDiff$padj>=pThresh&padGlm<pThresh)]<- col3
plot(glmIntExRef$table[,"logFC"], -1*log(padGlm, base=10), col=0, pch=16, 
    xlab="FC (log2)", ylab="GLM p-value (-log10)")
points(glmIntExRef$table[cols=="black","logFC"], -1*log(padGlm[cols=="black"], 
    base=10), col=cols[cols=="black"], pch=16)
points(glmIntExRef$table[cols==col1,"logFC"], -1*log(padGlm[cols==col1], 
    base=10), col=cols[cols==col1], pch=16)
points(glmIntExRef$table[cols==col2,"logFC"], -1*log(padGlm[cols==col2], 
    base=10), col=cols[cols==col2], pch=16)
points(glmIntExRef$table[cols==col3,"logFC"], -1*log(padGlm[cols==col3], 
    base=10), col=cols[cols==col3], pch=16)
abline(h=2, col="red", lwd=2)
legend("topleft", legend=c("DESeq2 and GLM p-values < 0.01", 
    "Only DESeq2 p-value < 0.01", "Only GLM p-value < 0.01"), 
    fil=c(col1, col2, col3))
text(-7.8,2.5,"Y = 2",col="red", cex=1.5)
title(main = "(B)")
```

**Fig. S3.** Volcano plots based on p-values and foldchange values obtained from (A) IntEREst-DESeq2 and (B) IntEREst-edgeR. The common and the unique discovered significantly more/less retained introns have been labeled and described in the legend.

## Comparing IntEREst-DESeq2 to IntEREst-DEXSeq

Here we compare the significantly more and less retained introns discovered by running IntEREst-DEXSeq on the MDS data and compare to the results obtained from IntEREst-DESeq2. Running the following script is however time consuming; you can skip this script and continue.

```
# Time demanding
library(BiocParallel)
library(DEXSeq)
load(file="./mdsRefObj.rda")
mdsRefObjInt<- mdsRefObj[rowData(mdsRefObj)[,"int_ex"]=="intron",]
dexObjMdsRefObjInt<- DEXSeqDataSet( 
    countData=counts(mdsRefObjInt), 
    sampleData=as.data.frame(colData(mdsRefObjInt)),
    design= ~ sample + exon + test_ctrl:exon,
    featureID=as.character(1:nrow(counts(mdsRefObjInt))), 
    groupID=as.character(rowData(mdsRefObjInt)[,"collapsed_transcripts_id"]))
save(dexObjMdsRefObjInt, file="dexObjMdsRefObjInt.rda")
#Filtering transcripts with introns that feature low read counts
sfMdsRefObjInt<- estimateSizeFactors(dexObjMdsRefObjInt)
feCntMdsRefObjInt<- rowMeans(featureCounts(sfMdsRefObjInt, TRUE))
maxRetTr<- tapply(feCntMdsRefObjInt, 
    as.character(rowData(mdsRefObjInt)[,"collapsed_transcripts_id"]), 
    max)
inTr<- names(maxRetTr)[which(as.numeric(maxRetTr)>1)]
mdsFilBool<- rowData(mdsRefObjInt)[,"collapsed_transcripts_id"] %in% inTr
mdsRefObjFilDsInt<- mdsRefObjInt[mdsFilBool,]
save(mdsRefObjFilDsInt, file="mdsRefObjFilDsInt.rda")
save(inTr, file="inTr.rda")

#DEXSeq test. Run silently
capture.output( dexseqFilIntRef<- DEXSeqIntEREst(x=mdsRefObjFilDsInt, 
    design= ~ sample + intron + test_ctrl:intron,
    reducedModel = ~ sample + intron, fitExpToVar="test_ctrl", 
    intExCol="int_ex", geneIdCol="collapsed_transcripts", silent=TRUE, 
    bpparam=SnowParam(workers=30)), file='NUL')
save(dexseqFilIntRef, file="./dexseqFilIntRef.rda")
```

The following script plots the distributuion of pvalues to the log fold change in the results obtained from IntEREst-DESeq2 and IntEREst-DEXSeq. It also shows the overlapping and uniquely discovered differentialy reatined introns by the two methods.

```
load(file="./mdsRefObj.rda")
load("./dexseqFilIntRef.rda")
load("./mdsRefObjFilDsInt.rda")
load("./inTr.rda")
# Get index of filtered data
mdsRefObjInt<- mdsRefObj[rowData(mdsRefObj)$int_ex=="intron",]
indDex<- which(rowData(mdsRefObjInt)[,"collapsed_transcripts_id"]
    %in% inTr)
indDex<-indDex[which(rowData(mdsRefObjFilDsInt)[,"int_ex"]=="intron")]
indDds<- which(rowData(mdsRefIntExObj)[,"int_ex"]=="intron")


cols<- rep("black", length(indDex))
indSigDex<-indDex[which(dexseqFilIntRef$pvalue<pThresh)]
indSigDds<-indDds[which(ddsDiff$padj<pThresh)]

cols<- rep("black", length(indDex))
cols[which(dexseqFilIntRef$pvalue<pThresh)]<- col3
cols[which(indDex%in%indSigDds)]<- col1
cols[which(indDex %in% indSigDds[which(!(indSigDds%in%indSigDex))])]<- col2
par(mfrow=c(2,1))
pointsize=.7
plot(dexseqFilIntRef$log2fold_test_ctrl, 
    -1*log(dexseqFilIntRef$pvalue, base=10), col=0, pch=16, xlab="FC (log2)", 
    ylab="DEXSeq p-value (-log10)", cex=pointsize)
points(dexseqFilIntRef[which(cols=="black"),"log2fold_test_ctrl"], 
    -1*log(dexseqFilIntRef$pvalue[which(cols=="black")], base=10), 
    col=cols[which(cols=="black")], pch=16, cex=pointsize)
points(dexseqFilIntRef[which(cols==col1),"log2fold_test_ctrl"], 
    -1*log(dexseqFilIntRef$pvalue[which(cols==col1)], base=10), 
    col=cols[which(cols==col1)], pch=16, cex=pointsize)
points(dexseqFilIntRef[which(cols==col2),"log2fold_test_ctrl"], 
    -1*log(dexseqFilIntRef$pvalue[which(cols==col2)], base=10), 
    col=cols[which(cols==col2)], pch=16, cex=pointsize)

abline(h=2, col="red", lwd=2)
legend("topleft", legend=c("DESeq2 and DEXSeq p-values < 0.01", 
    "Only DESeq2 p-value < 0.01"), 
    fil=c(col1, col2))
text(38,6,"Y = 2",col="red", cex=1.5)
title(main = "(A)")

plot(dexseqFilIntRef$log2fold_test_ctrl, 
    -1*log(dexseqFilIntRef$pvalue, base=10), col=0, pch=16, xlab="FC (log2)", 
    ylab="DEXSeq p-value (-log10)", cex=pointsize)
points(dexseqFilIntRef[which(cols=="black"),"log2fold_test_ctrl"], 
    -1*log(dexseqFilIntRef$pvalue[which(cols=="black")], base=10), 
    col=cols[which(cols=="black")], pch=16, cex=pointsize)
points(dexseqFilIntRef[which(cols==col1),"log2fold_test_ctrl"], 
    -1*log(dexseqFilIntRef$pvalue[which(cols==col1)], base=10), 
    col=cols[which(cols==col1)], pch=16, cex=pointsize)
points(dexseqFilIntRef[which(cols==col3),"log2fold_test_ctrl"], 
    -1*log(dexseqFilIntRef$pvalue[which(cols==col3)], base=10), 
    col=cols[which(cols==col3)], pch=16, cex=pointsize)
abline(h=2, col="red", lwd=2)
legend("topleft", legend=c("DESeq2 and DEXSeq p-values < 0.01", 
    "Only DEXSeq p-value < 0.01"), 
    fil=c(col1, col3))
text(38,6,"Y = 2",col="red", cex=1.5)
title(main = "(B)")
```

**Fig. S4.** Volcano plots based on p-values and foldchange values obtained from (A) IntEREst-DESeq2 and (B) IntEREst-DEXSeq. The common and the unique discovered significantly more/less retained introns have been labeled and described in the legend.

## Comparing IntEREst-DESeq2 results to the retained introns discovered by IRFInder

The IRFinder scripts are stated in `./IRFinderScripts.sh` and the downstream differentiation analysis of the IRFinder using DESeq2 are included in `./IRFinderDESeq2.R`. Both of these files are located in the `scripts` folder in the GitHub repository. The results of the analysis are also available in the repository as R objects. Here, we load load the required objects in R and compare the results obtained by IntEREst-DESeq2 to the IRFinder.

```
load("./irfDiff.rda")
load("./mdsRefIntExObj.rda")
load("./ddsDiff.rda")
library(GenomicRanges)
mdsRefDdsObjInt<- mdsRefIntExObj[rowData(mdsRefIntExObj)$int_ex=="intron",]
mdsRefDdsObjIntGr<- GRanges(rowData(mdsRefDdsObjInt)$chr,
    IRanges(rowData(mdsRefDdsObjInt)$begin, 
        rowData(mdsRefDdsObjInt)$end))

irfChr<- paste("chr", gsub(".*/","",gsub(": *.*","",rownames(irfDiff))), 
    sep="")
irfBeg<-as.numeric(gsub(".*: *([0-9]+)[-+].*","\\1",rownames(irfDiff)))+1
irfEnd<- as.numeric(gsub(".*[ -]","", gsub(":[-+]$","", rownames(irfDiff))))

irfGr<- GRanges(irfChr, IRanges(irfBeg, irfEnd))


irfMdsRefObjIntMatch<- suppressWarnings(
    findOverlaps(irfGr, mdsRefDdsObjIntGr, type="equal") )

mdsRefDdsObjIntSel<- mdsRefDdsObjInt[subjectHits(irfMdsRefObjIntMatch),]
ddsDiffSel<- ddsDiff[subjectHits(irfMdsRefObjIntMatch),]
irfDiffSel<- irfDiff[queryHits(irfMdsRefObjIntMatch),]

cols<-rep("black", length(irfDiffSel$log2FoldChange))
cols[ddsDiffSel$padj<0.01&irfDiffSel$padj>=0.01]<- col2
cols[irfDiffSel$padj<0.01&ddsDiffSel$padj>=0.01]<- col3
cols[ddsDiffSel$padj<0.01&irfDiffSel$padj<0.01]<- col1
plot(irfDiffSel$log2FoldChange[cols=="black"], 
    ddsDiffSel$log2FoldChange[cols=="black"], col=cols[cols=="black"], 
    xlab="IRFinder", ylab="DESeq2", pch=16, cex=1,
    xlim=c(min(c(irfDiffSel$log2FoldChange, ddsDiffSel$log2FoldChange), 
            na.rm=TRUE), 
        max(c(irfDiffSel$log2FoldChange, ddsDiffSel$log2FoldChange),
            na.rm=TRUE)),
    ylim=c(min(c(irfDiffSel$log2FoldChange, ddsDiffSel$log2FoldChange), 
            na.rm=TRUE), 
        max(c(irfDiffSel$log2FoldChange, ddsDiffSel$log2FoldChange), 
            na.rm=TRUE)))
points(irfDiffSel$log2FoldChange[cols==col2], 
    ddsDiffSel$log2FoldChange[cols==col2], col=cols[cols==col2], pch=16, cex=1)
points(irfDiffSel$log2FoldChange[cols==col3], 
    ddsDiffSel$log2FoldChange[cols==col3], col=cols[cols==col3], pch=16, cex=1)
points(irfDiffSel$log2FoldChange[cols==col1], 
    ddsDiffSel$log2FoldChange[cols==col1], col=cols[cols==col1], pch=16, cex=1)
legend("topleft", legend=c("DESeq2 and IRFinder p-value < 0.01", 
    "Only DESeq2 p-value < 0.01", "Only IRFinder p-value < 0.01"), 
    fil=c(col1, col2, col3))
(lmRes<- lm(ddsDiffSel$log2FoldChange~irfDiffSel$log2FoldChange))
```

```
## 
## Call:
## lm(formula = ddsDiffSel$log2FoldChange ~ irfDiffSel$log2FoldChange)
## 
## Coefficients:
##               (Intercept)  irfDiffSel$log2FoldChange  
##                    0.1215                     0.6491
```

```
abline(lmRes, 
    col="red", lwd=2)
abline(a=0,b=1, col="red", lty=2, lwd=2)

text(-6.5,-8,"Y = X",col="red", cex=1.5)
text(-8,-4,"Y ~ X",col="red", cex=1.5)
```

**Fig. S5.** The log2 fold change of IR values using DESeq2 vs The log2 fold change of IR values using IRFinder. The overlapping retained introns (i.e. discovered by IRFinder and IntEREst) and the uniquely detected retained introns are labeled with different colours. The continuous red line is the Y=X line and the line labeled with Y~X was resulted by fitting a linear model to the data shown in the plot (*i.e.* Y= 0.64 X + 0.12).

## Comparing IntEREst-DESeq2 results to the IRs reported in the MDS study

Here we compare the significantly retained introns extracted from IntEREst-DESeq2 to those reported by V. Madan *et al.* (2015).

```
#Change this path if you prefer the temp files be copied elsewhere that the 
# working directory
tmpPath<- "./"
library(xlsx)
# Download supplementary data
download.file(url = 
"https://www.nature.com/article-assets/npg/ncomms/2015/150114/ncomms7042/extref/ncomms7042-s2.xls", 
    destfile = paste(tmpPath, "ncomms7042-s2.xls", sep="/"))
vikasDat<- read.xlsx(file="./ncomms7042-s2.xls", sheetIndex=1, startRow=3, 
    endRow=260, as.data.frame=TRUE, header=TRUE)

#Make GRanges object
vikasGr<- GRanges(vikasDat[,"Chromosome"], 
    IRanges(as.numeric(vikasDat[,"Intron.start"]), 
        as.numeric(vikasDat[,"Intron.end"])))


#Extract the indexes of significantly Up and Down retained introns for DEXSeq
indDexseq<-1:nrow(mdsRefObjInt)
pThresh<- 0.01

mdsRefObjIntGr<- GRanges(rowData(mdsRefObjInt)[,"chr"], 
    IRanges(as.numeric(rowData(mdsRefObjInt)[,"begin"]), 
        as.numeric(rowData(mdsRefObjInt)[,"end"])))

# Extract overlap of the results discovered by Madan et al. (2015) and the 
# reference used in IntEREst
vikasMdsRefObjIntMatch<-findOverlaps(vikasGr, mdsRefObjIntGr, 
    type="equal")
# Extract Madan et al. (2015) results that match the refernce used in IntEREst
vikasValidGr<- vikasGr[unique(queryHits(vikasMdsRefObjIntMatch)),] 

load("./ddsDiff.rda")

# Extract index of significantly more retained introns 
indUpMdsRefDdsObjInt<- which(ddsDiff$padj<pThresh & 
    ddsDiff$log2FoldChange>0)
mdsRefDdsObjIntUp<-mdsRefDdsObjInt[indUpMdsRefDdsObjInt,]
# Make GRanges of higher retained introns (detected by IntEREst-DESeq2
mdsRefDdsObjIntUpGr<- GRanges(rowData(mdsRefDdsObjIntUp)$chr,
    IRanges(rowData(mdsRefDdsObjIntUp)$begin, 
        rowData(mdsRefDdsObjIntUp)$end))

# Extract overlap of the 2 set of significantly higher retained results
vikasMdsRefObjIntUpMatch<-findOverlaps(vikasValidGr, mdsRefDdsObjIntUpGr, 
    type="equal")

# Percentage of IRs discovered by Madan et al. (2015) that were also discovered
# by IntEREst-DESeq2
100* table(rowData(mdsRefDdsObjIntUp)[unique(subjectHits(
        vikasMdsRefObjIntUpMatch)), "intron_type"])/
    table(rowData(mdsRefObjInt)[unique(subjectHits(vikasMdsRefObjIntMatch)),
        "intron_type"])
```

```
## 
##      U12   U12/U2       U2 
## 88.23529 80.00000 74.64789
```

```
# Number of IRs discovered by Madan et al. (2015), also discovered by 
# IntEREs-DESeq2
table(rowData(mdsRefObjInt)[unique(subjectHits(vikasMdsRefObjIntMatch)),
    "intron_type"])
```

```
## 
##    U12 U12/U2     U2 
##    136      5     71
```

```
# Number of additional IRs discovered by IntEREst-DESeq2
table(rowData(mdsRefDdsObjIntUp)[,"intron_type"])-
    table(rowData(mdsRefDdsObjIntUp)[unique(subjectHits(
        vikasMdsRefObjIntUpMatch)),"intron_type"])
```

```
## 
##    U12 U12/U2     U2 
##    149      0   1195
```

```
cols=rep("black", length(ddsDiff$padj))
cols[ddsDiff$padj<pThresh]<- col2
cols[indUpMdsRefDdsObjInt[unique(subjectHits(vikasMdsRefObjIntUpMatch))]]<- col1
cols[unique(subjectHits(vikasMdsRefObjIntMatch))[which(! unique(subjectHits(
    vikasMdsRefObjIntMatch)) %in% indUpMdsRefDdsObjInt[unique(subjectHits(
    vikasMdsRefObjIntUpMatch))])]]<- col3

pch=16
cex=.8
plot(ddsDiff$log2FoldChange, -1*log(ddsDiff$padj, base=10), 
    col=0, pch=pch, xlab="FC (log2)", ylab="p-value (-log10)")
points(ddsDiff$log2FoldChange[cols=="black"], 
    -1*log(ddsDiff$padj[cols=="black"], base=10), col=cols[cols=="black"], 
        pch=pch, cex=cex)
points(ddsDiff$log2FoldChange[cols==col2], -1*log(ddsDiff$padj[cols==col2],
    base=10), col=cols[cols==col2], pch=pch, cex=cex)
points(ddsDiff$log2FoldChange[cols==col1], -1*log(ddsDiff$padj[cols==col1],
    base=10), col=cols[cols==col1], pch=pch, cex=cex)
points(ddsDiff$log2FoldChange[cols==col3], -1*log(ddsDiff$padj[cols==col3],
    base=10), col=cols[cols==col3], pch=pch, cex=cex)
legend("topleft", legend=c("DESeq2 and Madan et al. < 0.01", 
    "Only DESeq2 p-value < 0.01", "Only Madan et al. < 0.01"), 
    fil=c(col1, col2, col3))
abline(h=2, col="red", lwd=2)
text(-8,3.5,"Y = 2",col="red", cex=1.5)
```

**Fig. S6.** Volcano plots based on p-values and foldchange values obtained from IntEREst-DESeq2. The common and uniquely discovered significantly retained introns by DESeq2 and/or by V. Madan *et al.* (2015) have been labeled with different colours.

The following scripts generates the Fig. 3 in the paper.

```
# Fig. 3
library(grid)
library(gridBase)
library(utils)
library(xlsx)
library(GenomicRanges)
library(VennDiagram)

### DESeq2
load("./mdsRefObj.rda")
load("./mdsExRefObj.rda")
load("./mdsRefIntExObj.rda")
load("./ddsDiff.rda")
mdsRfObjIntBool<-rowData(mdsRefObj)$int_ex=="intron"
mdsRefObjInt<- mdsRefObj[mdsRfObjIntBool,]

mdsRefDdsObjInt<- mdsRefIntExObj[rowData(mdsRefIntExObj)$int_ex=="intron",]
mdsRefDdsObjIntGr<- GRanges(rowData(mdsRefDdsObjInt)$chr,
    IRanges(rowData(mdsRefDdsObjInt)$begin, 
        rowData(mdsRefDdsObjInt)$end))

pThresh<- 0.01

indUpMdsRefDdsObjInt<- which(ddsDiff$padj<pThresh & 
    ddsDiff$log2FoldChange>0)
mdsRefDdsObjIntUp<-mdsRefDdsObjInt[indUpMdsRefDdsObjInt,]
mdsRefDdsObjIntUpGr<- GRanges(rowData(mdsRefDdsObjIntUp)$chr,
    IRanges(rowData(mdsRefDdsObjIntUp)$begin, 
        rowData(mdsRefDdsObjIntUp)$end))
indDnMdsRefDdsObjInt<- which(ddsDiff$padj<pThresh & 
    ddsDiff$log2FoldChange<0)
mdsRefDdsObjIntDn<-mdsRefDdsObjInt[indDnMdsRefDdsObjInt,]
mdsRefDdsObjIntDnGr<- GRanges(rowData(mdsRefDdsObjIntDn)$chr,
    IRanges(rowData(mdsRefDdsObjIntDn)$begin, 
        rowData(mdsRefDdsObjIntDn)$end))

###EdgeR
glmIntExRef<- glmInterest(mdsRefIntExObj,
    sampleAnnoCol="test_ctrl", sampleAnnotation=c("ctrl","test"),
    geneIdCol= "collapsed_transcripts_id", silent=TRUE, disp="common", 
    design=model.matrix(~test_ctrl+test_ctrl:intronExon, 
    data= colData(tmpObj)),
    contrast=c(0,0,-1,1)
    )
save(glmIntExRef, file="./glmIntExRef.rda")

padGlm<- p.adjust(glmIntExRef$table$PValue, method="BH")
lfcGlm<-glmIntExRef$table$logFC

pThresh<- 0.01

mdsRefEdgRObjInt<- mdsRefObj[rowData(mdsRefObj)$int_ex=="intron",]
mdsRefEdgRObjIntGr<- GRanges(rowData(mdsRefEdgRObjInt)$chr,
    IRanges(rowData(mdsRefEdgRObjInt)$begin, 
        rowData(mdsRefEdgRObjInt)$end))


indUpMdsRefEdgRObjInt<- which(padGlm<pThresh & 
    lfcGlm>0)
mdsRefEdgRObjIntUp<-mdsRefEdgRObjInt[indUpMdsRefEdgRObjInt,]
mdsRefEdgRObjIntUpGr<- GRanges(rowData(mdsRefEdgRObjIntUp)$chr,
    IRanges(rowData(mdsRefEdgRObjIntUp)$begin, 
        rowData(mdsRefEdgRObjIntUp)$end))
indDnMdsRefEdgRObjInt<- which(padGlm<pThresh & 
    lfcGlm<0)
mdsRefEdgRObjIntDn<-mdsRefEdgRObjInt[indDnMdsRefEdgRObjInt,]
mdsRefEdgRObjIntDnGr<- GRanges(rowData(mdsRefEdgRObjIntDn)$chr,
    IRanges(rowData(mdsRefEdgRObjIntDn)$begin, 
        rowData(mdsRefEdgRObjIntDn)$end))
#### DEXSeq VS Deseq2

load("./dexseqFilIntRef.rda")
load("./mdsRefObjFilDsInt.rda")

pThresh<- 0.01

# Extract the indexes of significantly Up and Down retained introns for DESEq2
indxUpMdsRefDdsObjInt<- which(mdsRfObjIntBool)[which(ddsDiff$padj<pThresh & 
    ddsDiff$log2FoldChange>0)]
indxDnMdsRefDdsObjInt<- which(mdsRfObjIntBool)[which(ddsDiff$padj<pThresh & 
    ddsDiff$log2FoldChange<0)]

#Extract the indexes of significantly Up and Down retained introns for DEXSeq

indUpMdsRefObjFilDsInt<- (which(mdsFilBool)[which(mdsFilIntBool)])[
    which(dexseqFilIntRef$padj<pThresh & dexseqFilIntRef$log2fold_test_ctrl>0)]
indDnMdsRefObjFilDsInt<- (which(mdsFilBool)[which(mdsFilIntBool)])[
    which(dexseqFilIntRef$padj<pThresh & dexseqFilIntRef$log2fold_test_ctrl<0)]

### IRFinder
#####irfinder
load("./irfDiff.rda")

mdsRefObjInt<- mdsRefObj[rowData(mdsRefObj)$int_ex=="intron",]

irfChr<- paste("chr", gsub(".*/","",gsub(": *.*","",rownames(irfDiff))), 
    sep="")
irfBeg<-as.numeric(gsub(".*: *([0-9]+)[-+].*","\\1",rownames(irfDiff)))+1
irfEnd<- as.numeric(gsub(".*[ -]","", gsub(":[-+]$","", rownames(irfDiff))))

irfGr<- GRanges(irfChr, IRanges(irfBeg, irfEnd))

#Extract the indexes of significantly Up and Down retained introns for DEXSeq
pThresh<- 0.01
indIrfUp<- which(irfDiff$padj<pThresh & irfDiff$log2FoldChange>0)
indIrfDn<- which(irfDiff$padj<pThresh & irfDiff$log2FoldChange<0)
irfUpGr<-irfGr[indIrfUp,]
irfDnGr<-irfGr[indIrfDn,]

mdsRefObjIntGr<- GRanges(rowData(mdsRefObjInt)$chr,
    IRanges(rowData(mdsRefObjInt)$begin, 
        rowData(mdsRefObjInt)$end))

irfUpMdsRefDdsObjIntUpMatch<- suppressWarnings(
    findOverlaps(irfUpGr, mdsRefDdsObjIntUpGr, type="equal") )
irfUpMdsRefDdsObjIntMatch<- suppressWarnings(
    findOverlaps(irfUpGr, mdsRefDdsObjIntGr, type="equal") )
irfMdsRefDdsObjIntUpMatch<- suppressWarnings(
    findOverlaps(irfGr, mdsRefDdsObjIntUpGr, type="equal") )

#### V.  Madan et al. (2015)
# Downloading and loading retained introns reported by V. Madan et al. (2015)
download.file(url = 
"https://www.nature.com/article-assets/npg/ncomms/2015/150114/ncomms7042/extref/ncomms7042-s2.xls", 
    destfile = "./ncomms7042-s2.xls")
vikasDat<- read.xlsx(file="./ncomms7042-s2.xls", sheetIndex=1, startRow=3, 
    endRow=260, as.data.frame=TRUE, header=TRUE)

vikasGr<- GRanges(vikasDat[,"Chromosome"], 
    IRanges(as.numeric(vikasDat[,"Intron.start"]), 
        as.numeric(vikasDat[,"Intron.end"])))


#Extract the indexes of significantly Up and Down retained introns for DEXSeq
indDexseq<-1:nrow(mdsRefObjInt)
pThresh<- 0.01

vikasMdsRefObjIntMatch<-findOverlaps(vikasGr, mdsRefObjIntGr, 
    type="equal")
# Extract all results that match the refernce used in IntEREst
vikasValidGr<- vikasGr[unique(queryHits(vikasMdsRefObjIntMatch)),] 
vikasMdsRefObjIntUpMatch<-findOverlaps(vikasValidGr, mdsRefDdsObjIntUpGr, 
    type="equal")
#Extract the indexes of significantly more retained introns for DEXSeq
pThresh<- 0.01


#### plots
aVenn<-draw.pairwise.venn(area1=length(indUpMdsRefDdsObjInt), 
    area2=length(indUpMdsRefEdgRObjInt),
    cross.area=length(intersect(indUpMdsRefDdsObjInt, indUpMdsRefEdgRObjInt)), 
    category = c("DESeq2\nup    ", "edgeR\n  up"), 
    fill = c("yellow", "red"), cat.pos=c(220,140), 
    cat.just=list(c(0.8,.9),c(0.1,0.6)), cat.cex=1,
    ext.line.lty=3, ext.line.lwd=.8, ext.dist= c(0, 0.05), 
    ext.length= rep(.8, 2), ind = FALSE)
bVenn<-draw.pairwise.venn(area1=length(indDnMdsRefDdsObjInt), 
    area2=length(indDnMdsRefEdgRObjInt),
    cross.area=length(intersect(indDnMdsRefDdsObjInt, indDnMdsRefEdgRObjInt)), 
    category = c("DESeq2\ndown", "edgeR\ndown"), 
    fill = c("yellow", "red"), cat.pos=c(45,45),
    ext.line.lty=3, ext.line.lwd=.8, ext.dist= c(0, 0.05), 
    ext.length= c(.9, 0.8), 
    cat.just=list(c(0.7,.9),c(0.7,0.8)), cat.cex=1, ind = FALSE)

cVenn<- draw.pairwise.venn(
    area1=length(indUpMdsRefObjFilDsInt), 
    area2=length(indxUpMdsRefDdsObjInt),
    cross.area=length(intersect(indUpMdsRefObjFilDsInt, indxUpMdsRefDdsObjInt)), 
    category = c("DEXSeq", "DESeq2"), 
    cat.pos=c(225, 135), cat.just=list(c(0.45,1.7),c(.6,1.9)), cat.cex=1, 
    ext.line.lty=3, ext.line.lwd=.8, ext.dist= c(0, 0.05), 
    ext.length= c(.9, 0.8),
    fill = c("green", "yellow"), ind = FALSE)

dVenn<- draw.pairwise.venn(
    area1=length(indDnMdsRefObjFilDsInt), 
    area2=length(indxDnMdsRefDdsObjInt),
    cross.area=length(intersect(indDnMdsRefObjFilDsInt, indxDnMdsRefDdsObjInt)), 
    category = c("DEXSeq", "DESeq2"), 
    cat.pos=c(225, 220), cat.just=list(c(0.45,1.7),c(.8,.7)), cat.cex=1, 
    ext.line.lty=3, ext.line.lwd=.8, ext.dist= c(0, 0.05), 
    ext.length= c(.9, 0.8),
    fill = c("green", "yellow"), ind = FALSE)
eVenn<- draw.pairwise.venn(
    area1=length(unique(queryHits(irfUpMdsRefDdsObjIntMatch))), 
    area2=length(unique(subjectHits(irfMdsRefDdsObjIntUpMatch))),
    cross.area=length(unique(queryHits(irfUpMdsRefDdsObjIntUpMatch))), 
    category = c("IR-\nFinder", "DESeq2"), 
    cat.pos=c(45, 45), cat.just=list(c(0.6,.8),c(.6,1.1)), cat.cex=1, 
    ext.line.lty=3, ext.line.lwd=.8, ext.dist= c(0, 0.05), 
    ext.length= c(.9, 0.8),
    fill = c("green", "yellow"), ind = FALSE)
fVenn<- draw.pairwise.venn(
    area1=length(unique(queryHits(
        vikasMdsRefObjIntMatch))), 
    area2=length(mdsRefDdsObjIntUpGr),
    cross.area=length(unique(queryHits(vikasMdsRefObjIntUpMatch))), 
    category = c("MDS", "DESeq2"), 
    cat.pos=c(45, 45), cat.just=list(c(0.5,.8),c(.55,1.3)), cat.cex=1,
    ext.line.lty=3, ext.line.lwd=.8, ext.dist= c(0, 0.05), 
    ext.length= c(.9, 0.8),fill = c("blue", "yellow"), 
    ind = FALSE)

tiff("./figures/fig3.tif", width=1200, height=800, res=300, pointsize=5)
par(lwd=.5)
par(cex=1)
par(cex.axis=1)
par(cex.lab=1)
par(mfrow=c(3,1))
par(mar=c(2,2,2,2))
layout(matrix(1:6, 2, byrow = FALSE))
frame()
vps <- baseViewports()
pushViewport(vps$inner, vps$figure, vps$plot)
grid.draw(aVenn)
title("(A)", outer=FALSE)
popViewport(3)

frame()
vps <- baseViewports()
pushViewport(vps$inner, vps$figure, vps$plot)
grid.draw(bVenn)
title("(B)", outer=FALSE)
popViewport(3)

frame()
vps <- baseViewports()
pushViewport(vps$inner, vps$figure, vps$plot)
grid.draw(cVenn)
title("(C)", outer=FALSE)
popViewport(3)

frame()
vps <- baseViewports()
pushViewport(vps$inner, vps$figure, vps$plot)
grid.draw(dVenn)
title("(D)", outer=FALSE)
popViewport(3)

frame()
vps <- baseViewports()
pushViewport(vps$inner, vps$figure, vps$plot)
grid.draw(eVenn)
title("(E)", outer=FALSE)
popViewport(3)

frame()
vps <- baseViewports()
pushViewport(vps$inner, vps$figure, vps$plot)
grid.draw(fVenn)
title("(F)", outer=FALSE)
popViewport(3)
dev.off()
```

# sensitivity analysis

We analyze the variations in the number of discovered significantly more/less retained introns caused by using various number of biological replicates and various sequencing library size.

## Sample size analysis

Here, we show the number of the discovered significantly higher and lower retained introns (comparing ZRSR2mut samples to controls) for various number of biological replicates. The following run is however time demanding. You can skip this stage.

```
# Time demanding
library(BiocParallel)
nSample=10
setNewVal<-FALSE
firstLoc<- getwd()
set.seed(10)
load("./mdsRefObj.rda")
load("./mdsExRefObj.rda")
mdsRefObjInt<- mdsRefObj[rowData(mdsRefObj)$int_ex=="intron",]
mdsRefIntExObj<- interestResultIntEx(intObj=mdsRefObjInt, exObj=mdsExRefObj, 
    mean.na.rm=TRUE, postExName="ex_junc" )
mdsRefDdsObjInt<- mdsRefIntExObj[rowData(mdsRefIntExObj)$int_ex=="intron",]
save(mdsRefDdsObjInt, file="./mdsRefDdsObjInt.rda")
indcntRes=c()
lenSamIndRes=c()
testIndRes=list()
ctrlIndRes=list()
deseqRes=list()
tmpRdaFolder<-"tmpNew/individual"
dir.create("tmpNew")
dir.create(tmpRdaFolder)

for(cnt in 1:8){
    print(cnt)
    combTest<-combn(1:8, cnt)
    combCtrl<-combn(9:16, cnt)
    expMat<-expand.grid(a = 1:ncol(combTest), b = 1:ncol(combCtrl))
    if(nrow(expMat)>nSample){
        samInd<- sample(1:nrow(expMat), nSample)
    } else {
        samInd<-(1:nrow(expMat))
    }
    for(indCnt in 1:length(samInd) ){
        print(paste(cnt, "/", 8, ",",indCnt,"/", length(samInd) ))
        deseqTmp<- deseqInterest(
            x=mdsRefIntExObj[,
                c(combTest[,expMat[samInd[indCnt],1]],
                    combCtrl[,expMat[samInd[indCnt],2]],
                    combTest[,expMat[samInd[indCnt],1]]+16,
                    combCtrl[,expMat[samInd[indCnt],2]]+16)],
            design=~test_ctrl+test_ctrl:intronExon,
            sizeFactor=rep(1,nrow(colData(mdsRefIntExObj[,
                c(combTest[,expMat[samInd[indCnt],1]],
                    combCtrl[,expMat[samInd[indCnt],2]],
                    combTest[,expMat[samInd[indCnt],1]]+16,
                    combCtrl[,expMat[samInd[indCnt],2]]+16)]))),
            contrast=list("test_ctrltest.intronExonintron",
                "test_ctrlctrl.intronExonintron"),
            parallel=TRUE,
            BPPARAM = SnowParam(workers=10))

            indcntRes<- c(indcntRes, indCnt)
            lenSamIndRes<- c(lenSamIndRes, length(samInd))
            testIndRes<- c(testIndRes, 
                list(combTest[,expMat[samInd[indCnt],1]]))
            ctrlIndRes<-c(ctrlIndRes, 
                list(combCtrl[,expMat[samInd[indCnt],2]]))

            save(deseqTmp, 
                file=paste(tmpRdaFolder, "/deseqTmp_", cnt, "_", indCnt,
                    ".rda", sep="") )
            save(lenSamIndRes, 
                file=paste(tmpRdaFolder, "/lenSamIndRes_", cnt, "_", indCnt,
                    ".rda", sep="") )
            save(testIndRes, 
                file=paste(tmpRdaFolder, "/testIndRes_", cnt, "_", indCnt,
                    ".rda", sep="") )
            save(ctrlIndRes, 
                file=paste(tmpRdaFolder, "/ctrlIndRes_", cnt, "_", indCnt,
                    ".rda", sep="") )
}
}

indsNames<- expand.grid(1:10, 1:8)[,c(2,1)]
indsNames<- indsNames[1:71,]

upIntCnt<- c()
dnIntCnt<- c()

lenSam<- sapply(testIndRes, length)
for(i in 1:nrow(indsNames)){
    print(paste(i, "/", nrow(indsNames)))
    desFile<- paste(
        tmpRdaFolder, "/deseqTmp_",
        indsNames[i,1],"_",indsNames[i,2],".rda", sep="")

    load(desFile)
    pThresh<- 0.01
    upIntCnt<-c(upIntCnt,  length(which(deseqTmp$padj< pThresh & 
        deseqTmp$log2FoldChange>0)) )
    dnIntCnt<-c(dnIntCnt, length(which(deseqTmp$padj< pThresh & 
        deseqTmp$log2FoldChange<0)))
}
save(upIntCnt, file="./upIntCnt.rda")
save(dnIntCnt, file="./dnIntCnt.rda")
save(lenSam, file="./lenSam.rda")
```

## Read coverage analysis

We study the variations in the number of the discovered significantly more and less retained introns (comparing ZRSR2mut samples to controls) affected by considering various number of mapped reads (or library size). For this analysis 5million, 10million, and … up to 50million reads are distributed proportionally based on the retention of the introns and the exon junction levels (their truncated values are considered). Based on these values, by using the `DESeqIntEREst()` function significantly more and less retained introns (when comparing ZRSR2mut to the controls) are extracted similar to the analysis mentioned above. The following run is however time demanding. You can skip this stage.

```
# Time demanding
load("./mdsRefDdsObjInt.rda")
colSums(counts(mdsRefDdsObjInt))[1:16]+colSums(counts(mdsRefDdsObjInt))[17:32]


# Average number of mapped reads across the samples
(avgReadCover<- mean(colSums(counts(mdsRefDdsObjInt))[1:16]+
    colSums(counts(mdsRefDdsObjInt))[17:32]))

# Max number of mapped reads across the samples
max(colSums(counts(mdsRefDdsObjInt))[1:16]+
    colSums(counts(mdsRefDdsObjInt))[17:32])

# Minimum number of mapped reads across the samples
min(colSums(counts(mdsRefDdsObjInt))[1:16]+
    colSums(counts(mdsRefDdsObjInt))[17:32])


firstLoc<- getwd()

tmpRdaFolder<-"tmpNew/individualReadDep"
dir.create(tmpRdaFolder, recursive = TRUE)
setwd(tmpRdaFolder)
matTmp1<- counts(mdsRefDdsObjInt)[,1:16]
matTmp2<- counts(mdsRefDdsObjInt)[,17:32]
colnames(matTmp2)<- colnames(matTmp1)
cntMatTmp<- rbind(matTmp1, 
    matTmp2)
sumMat<- cbind(matrix(rep(as.numeric(colSums(cntMatTmp)), 
    nrow(matTmp1)), ncol=ncol(matTmp1), byrow=TRUE ),
    matrix(rep(as.numeric(colSums(cntMatTmp)), 
    nrow(matTmp1)), ncol=ncol(matTmp1), byrow=TRUE ))
rDep<- seq(5000000, by=5000000, length.out= 10)
library(IntEREst)
library(BiocParallel)

for(cnt in 1:length(rDep)){
    print(paste(cnt, length(rDep), sep="/"))
        mdsTmp<- mdsRefDdsObjInt
        mdsTmp@assays[[1]]<-trunc((rDep[cnt]*counts(mdsRefDdsObjInt))/sumMat) 
        deseqTmp<- deseqInterest(
            x=mdsTmp,
            design=~test_ctrl+test_ctrl:intronExon,
            sizeFactor=rep(1,nrow(colData(mdsTmp))),
            contrast=list("test_ctrltest.intronExonintron",
                "test_ctrlctrl.intronExonintron"),
            parallel=TRUE,
            BPPARAM = SnowParam(workers=20))

            save(deseqTmp, 
                file=paste("deseqTmp_", cnt,
                    ".rda", sep="") )
}

setwd(firstLoc)
pThresh<- .01
countsMat<- matrix(0, nrow=0, ncol=3)
for(cnt in 1:length(rDep)){
    load(file=paste(tmpRdaFolder, "/deseqTmp_", cnt,
        ".rda", sep=""))
    countsMat<- rbind(countsMat, 
        c( 
            length(which(deseqTmp$log2FoldChange>0 & 
                deseqTmp$padj<pThresh)),
            length(which(deseqTmp$log2FoldChange<0 & 
                deseqTmp$padj<pThresh)),
            length(which((deseqTmp$log2FoldChange>0 | 
                deseqTmp$log2FoldChange<0) & 
                deseqTmp$padj<pThresh))
        )
    )
}


desDif<-deseqInterest(
            x=mdsRefDdsObjInt,
            design=~test_ctrl+test_ctrl:intronExon,
            sizeFactor=rep(1,nrow(colData(mdsRefDdsObjInt))),
            contrast=list("test_ctrltest.intronExonintron",
                "test_ctrlctrl.intronExonintron"),
            parallel=TRUE,
            BPPARAM = SnowParam(workers=2))
save(desDif, file="./desDif.rda")

countsMat<- rbind(countsMat, 
    c( 
        length(which(desDif$log2FoldChange>0 & 
            desDif$padj<pThresh)),
        length(which(desDif$log2FoldChange<0 & 
            desDif$padj<pThresh)),
        length(which((desDif$log2FoldChange>0 | 
            desDif$log2FoldChange<0) & 
            desDif$padj<pThresh))
    )
)
save(countsMat, file="./countsMat.rda")
colnames(countsMat)<- 
    c("more_retained", "less_retained", "more_or_less_retained")
rownames(countsMat)<- c(rDep/1000000, "~60")
save(countsMat, file="./countsMat.rda")
```

The following script generates the figure *Fig. 4* in the paper.

```
load( file="./upIntCnt.rda")
load( file="./dnIntCnt.rda")
load( file="./lenSam.rda")
load(file="./countsMat.rda")
rDep<- seq(5000000, by=5000000, length.out= 10)
rownames(countsMat)<- c(rDep/1000000, "~60")
(avgReadCover<- mean(colSums(counts(mdsRefDdsObjInt))[1:16]+
    colSums(counts(mdsRefDdsObjInt))[17:32]))
#[1] 59533214

tiff("./figures/fig4.tif", width=1200, height=750, res=300, pointsize=6)
par(lwd=.7)
par(cex=1)
par(cex.axis=1)
par(cex.lab=1)
par(mfrow=c(3,1))
par(mar=c(3.6,3.6,1.9,1.2))
par(mgp = c(2, .7, 0))
layout(matrix(c(1,2,3,3),nrow=2, byrow=FALSE))

boxplotList<- tapply(upIntCnt,lenSam, c)
boxplotList<- boxplotList[-1]
boxplot(boxplotList, ylab="# of more retained introns", 
    xlab="# of biological replicates")
points(tapply(upIntCnt,lenSam, median)[-1], col=2, lwd=1, type="b", pch=16, 
    cex=1.4)
title(main = "(A)")

boxplotList<- tapply(dnIntCnt,lenSam, c)[-1]
boxplot(boxplotList, ylab="# of less retained introns", 
    xlab="# of biological replicates")
points(tapply(dnIntCnt,lenSam, median)[-1], col=2, lwd=1, type="b", pch=16, 
    cex=1.4)
title(main = "(B)")

par(cex.axis=.9)

plot(c(rDep/1000000, 60), countsMat[,"more_or_less_retained"], 
    type="b", pch=NA, col=NA, 
    ylim=c(0, max(countsMat)), xaxt='n', ylab="# of discovered introns", 
    xlab="Introns read coverage (million reads)")
points(rDep/1000000, countsMat[1:10,"more_or_less_retained"], 
    type="b", pch=23, col="purple")
points(avgReadCover/1000000, countsMat[11,"more_or_less_retained"], 
    type="p", pch=23, col="purple", bg="purple")
points(rDep/1000000, countsMat[1:10,"more_retained"], 
    type="b", pch=24, col="red")
points(avgReadCover/1000000, countsMat[11,"more_retained"], 
    type="p", pch=24, col="red", bg="red")
points(rDep/1000000, countsMat[1:10,"less_retained"], 
    type="b", pch=25, col="blue")
points(avgReadCover/1000000, countsMat[11,"less_retained"], 
    type="p", pch=25, col="blue", bg="blue")
axis(1, at=c(rDep,avgReadCover)/1000000 , labels=rownames(countsMat))
legend("topleft", legend=c("Sig. more/less retained", "Sig. more retained", 
    "Sig. less retained"), pch=c(23,24,25), col=c("purple", "red", "blue"), 
    box.lwd=NA, bty = "n")
title(main = "(C)")
dev.off()
```

# Differential retention analysis of Maize data

We ran analysis similar to those which we ran on MDS data, on a Maize data constructed of 12 samples, 6 of which featured mutation in RGH3 gene (orthologous to the human ZRSR2) and 6 other control samples:

```
# Build references with collapsed and uncollapsed exons
ensMaizeCollRef<- referencePrepare (sourceBuild="biomaRt", 
    biomart="plants_mart", biomartHost="plants.ensembl.org",
    biomartDataset="zmays_eg_gene", 
    circSeqs=GenomicFeatures::DEFAULT_CIRC_SEQS,
    ignore.strand=FALSE, addCollapsedTranscripts=TRUE,
    collapseExons=TRUE)

ensMaizeUncollRef<- referencePrepare (sourceBuild="biomaRt", 
    biomart="plants_mart", biomartHost="plants.ensembl.org",
    biomartDataset="zmays_eg_gene", 
    circSeqs=GenomicFeatures::DEFAULT_CIRC_SEQS,
    ignore.strand=TRUE, 
    collapseExons=FALSE, 
    addCollapsedTranscripts=FALSE)

# Select unique exons and group exons of overlapping transcripts by 
# assigning similar IDs
ensMaizeExDf<- unionRefTr(referenceChr= ensMaizeUncollRef[,"chr"], 
    referenceBegin= as.numeric(ensMaizeUncollRef[,"begin"]), 
    referenceEnd= as.numeric(ensMaizeUncollRef[,"end"]), 
    referenceTr=as.character(ensMaizeUncollRef[,"transcript_id"]),
    referenceIntronExon=ensMaizeUncollRef[,"int_ex"],
    intronExon="exon",
    silent=FALSE)

# Build DNA sequence data, i.e. A DNAStringSet object
srcFiles<- paste(
"ftp://ftp.ncbi.nlm.nih.gov/genomes/all/GCF/000/005/005/",
"GCF_000005005.2_B73_RefGen_v4/GCF_000005005.2_B73_RefGen_v4_assembly_",
"structure/Primary_Assembly/assembled_chromosomes/FASTA/chr", 1:10, ".fna.gz",
    sep="")
destFiles<- paste("./chr",1:10, ".fna.gz",  sep="")
lapply(1:10, 
    function(x)download.file(url=srcFiles[x], destfile=destFiles[x]))

lapply(dir(".", "*.gz"), R.utils::gunzip)
library(BSgenome)
forgeSeqFiles(seqnames=as.character(1:10),
    prefix="./chr", 
    suffix=".fna", ondisk_seq_format="2bit", verbose=TRUE)
bsMaizeB73<- import("./single_sequences.2bit")

ensMaizeCollRefFil<- 
    ensMaizeCollRef[
        ensMaizeCollRef$chr %in% paste("chr",c(1:10, "Mt", "Pt"),sep=""),]

# Correct strand information
ensMaizeCollRefFil$strand="*"

# annotate u12 introns

refseqAnnoMat<- annotateU12(
    pwmU12U2= list(
        pwmU12db[[1]][,11:17],
        pwmU12db[[2]],
        pwmU12db[[3]][,38:40],
        pwmU12db[[4]][,11:17],
        pwmU12db[[5]][,38:40]),
    pwmSsIndex= list(
        indexDonU12=1, 
        indexBpU12=1, 
        indexAccU12=3, 
        indexDonU2=1, 
        indexAccU2=3), 
    referenceChr= gsub("^chr","",ensMaizeCollRefFil[,"chr"]), 
    referenceBegin= as.numeric(ensMaizeCollRefFil[,"begin"]), 
    referenceEnd= as.numeric(ensMaizeCollRefFil[,"end"]), 
    referenceIntronExon= as.character(ensMaizeCollRefFil[,"int_ex"]),
    intronExon= "intron",
    matchWindowRelativeUpstreamPos= c(NA,-29,NA,NA,NA),
    matchWindowRelativeDownstreamPos= c(NA,-9,NA,NA,NA), 
    minMatchScore= c( rep("80%", 2), "40%", "80%",  "40%"), 
    refGenome= bsMaizeB73, 
    setNaAs= "U2", 
    annotateU12Subtype= TRUE)

# Define locations to the bam files
outDir<-"/netapp/ali/Maize_ZRSR2/interestRun/intret/"
MDS_BAMFILES<- paste(
    c(
        "/netapp/ali/Maize_ZRSR2/mapping/SRR1282039",
        "/netapp/ali/Maize_ZRSR2/mapping/SRR1282040",
        "/netapp/ali/Maize_ZRSR2/mapping/SRR1282041",
        "/netapp/ali/Maize_ZRSR2/mapping/SRR1282042",
        "/netapp/ali/Maize_ZRSR2/mapping/SRR1282043",
        "/netapp/ali/Maize_ZRSR2/mapping/SRR1282044",
        "/netapp/ali/Maize_ZRSR2/mapping/SRR1282045",
        "/netapp/ali/Maize_ZRSR2/mapping/SRR1282046",
        "/netapp/ali/Maize_ZRSR2/mapping/SRR1282047",
        "/netapp/ali/Maize_ZRSR2/mapping/SRR1282048",
        "/netapp/ali/Maize_ZRSR2/mapping/SRR1282049",
        "/netapp/ali/Maize_ZRSR2/mapping/SRR1282050"),
    "accepted_hits.bam", sep="/")
names(MDS_BAMFILES)<-  gsub(".*/","", 
    gsub("/accepted_hits.bam","", MDS_BAMFILES))

# Intron retention run

bamChrNames<-c("NC_024459.2",
"NC_024460.2",
"NC_024461.2",
"NC_024462.2",
"NC_024463.2",
"NC_024464.2",
"NC_024465.2",
"NC_024466.2",
"NC_024467.2",
"NC_024468.2",
"NC_007982.1",
"NC_001666.2")
names(bamChrNames)<- paste("chr", c(1:10,"Mt","Pt"), sep="")
ensMaizeCollRefFilBamChr<-ensMaizeCollRefFil
ensMaizeCollRefFilBamChr$chr<- as.character(
    bamChrNames[as.character(ensMaizeCollRefFilBamChr$chr)])
for(i in 1:length(MDS_BAMFILES)){
    dir.create(paste(outDir, names(MDS_BAMFILES)[i],
        sep="/"), recursive = TRUE)
   interest(
        bamFileYieldSize=1000000,
        bamFile=MDS_BAMFILES[i],
        isPaired=TRUE,
        isPairedDuplicate=FALSE,
        isSingleReadDuplicate=NA,
        reference=ensMaizeCollRefFilBamChr,
        referenceGeneNames=
            ensMaizeCollRefFilBamChr[,"collapsed_transcripts_id"],
        referenceIntronExon=ensMaizeCollRefFilBamChr[,"int_ex"],
        repeatsTableToFilter= c(),
        junctionReadsOnly=c(FALSE),
        outFile=paste(outDir, names(MDS_BAMFILES)[i], 
            "interestRes.tsv", sep="/"),
        logFile=paste(outDir, names(MDS_BAMFILES)[i], 
            "log.txt", sep="/"),
        method=c("IntRet"),
        clusterNo=40,
        returnObj=FALSE, 
        scaleLength= TRUE, 
        scaleFragment= TRUE
    )
}

mzIntRefObj<-readInterestResults(
    resultFiles=paste(outDir, names(MDS_BAMFILES), 
            "interestRes.tsv", sep="/"), 
    sampleNames=names(MDS_BAMFILES), 
    sampleAnnotation=data.frame( 
        name=c("SRR1282039","SRR1282040","SRR1282041","SRR1282042", 
            "SRR1282043","SRR1282044","SRR1282045","SRR1282046","SRR1282047",
            "SRR1282048","SRR1282049","SRR1282050"),
        norm_rg3= c("Norm", "Norm", "rgh3", "rgh3", "Norm", "Norm", "rgh3",
            "rgh3", "Norm", "Norm", "rgh3", "rgh3"),
        roots_shoots=c("roots", "shoots", "roots", "shoots", "roots", "shoots",
            "roots", "shoots", "roots", "shoots", "roots", "shoots"),
        test_ctrl=c("ctrl", "ctrl", "test", "test", "ctrl", "ctrl", "test",
            "test", "ctrl", "ctrl", "test", "test")
    ), 
    commonColumns=1:ncol(ensMaizeCollRefFilBamChr), 
    freqCol=ncol(ensMaizeCollRefFilBamChr)+1, 
    scaledRetentionCol=ncol(ensMaizeCollRefFilBamChr)+2,
    scaleLength=TRUE, scaleFragment=TRUE, reScale=TRUE, 
    geneIdCol="collapsed_transcripts_id")

# update the object with the intron type (U12- or U2-type) annotations
mzIntRefObj<- updateRowDataCol(mzIntRefObj,  "intron_type", 
    refseqAnnoMatMz[,1])

# Exon-exon junction run
load(file="/netapp/ali/Maize_ZRSR2/ensMaizeExDf.rda")
outDir<- "/netapp/ali/Maize_ZRSR2/interestRun/exex/"
dir.create(outDir)

bamChrNames<-c("NC_024459.2",
"NC_024460.2",
"NC_024461.2",
"NC_024462.2",
"NC_024463.2",
"NC_024464.2",
"NC_024465.2",
"NC_024466.2",
"NC_024467.2",
"NC_024468.2",
"NC_007982.1",
"NC_001666.2")

names(bamChrNames)<- paste("chr", c(1:10,"Mt","Pt"), sep="")
ensMaizeExDfFilBamChr<- 
    ensMaizeExDf[
        ensMaizeExDf$chr %in% names(bamChrNames),]

ensMaizeExDfFilBamChr$chr<- as.character(
    bamChrNames[as.character(ensMaizeExDfFilBamChr$chr)])


for(i in 1:length(MDS_BAMFILES)){
    dir.create(paste(outDir, names(MDS_BAMFILES)[i],
        sep="/"), recursive = TRUE)
   interest(
        bamFileYieldSize=1000000,
        bamFile=MDS_BAMFILES[i],
        isPaired=TRUE,
        isPairedDuplicate=FALSE,
        isSingleReadDuplicate=NA,
        reference=ensMaizeExDfFilBamChr,
        referenceGeneNames=ensMaizeExDfFilBamChr[,"transcripts_id"],
        referenceIntronExon=ensMaizeExDfFilBamChr[,"int_ex"],
        repeatsTableToFilter= c(),
        junctionReadsOnly=TRUE,
        outFile=paste(outDir, names(MDS_BAMFILES)[i], 
            "interestRes.tsv", sep="/"),
        logFile=paste(outDir, names(MDS_BAMFILES)[i], 
            "log.txt", sep="/"),
        method=c("ExEx"),
        returnObj=FALSE, 
        scaleLength= FALSE, 
        scaleFragment= TRUE,
        bpparam= SnowParam(workers=50)
    )
}

mzExRefObj<-readInterestResults(
    resultFiles=paste(outDir, names(MDS_BAMFILES), 
            "interestRes.tsv", sep="/"), 
    sampleNames=names(MDS_BAMFILES), 
    sampleAnnotation=data.frame( 
        name=c("SRR1282039","SRR1282040","SRR1282041","SRR1282042", 
            "SRR1282043","SRR1282044","SRR1282045","SRR1282046","SRR1282047",
            "SRR1282048","SRR1282049","SRR1282050"),
        norm_rg3= c("Norm", "Norm", "rgh3", "rgh3", "Norm", "Norm", "rgh3",
            "rgh3", "Norm", "Norm", "rgh3", "rgh3"),
        roots_shoots=c("roots", "shoots", "roots", "shoots", "roots", "shoots",
            "roots", "shoots", "roots", "shoots", "roots", "shoots"),
        test_ctrl=c("ctrl", "ctrl", "test", "test", "ctrl", "ctrl", "test",
            "test", "ctrl", "ctrl", "test", "test")
    ), 
    commonColumns=1:ncol(ensMaizeExDf), freqCol=ncol(ensMaizeExDf)+1, 
    scaledRetentionCol=ncol(ensMaizeExDf)+2,
    scaleLength=FALSE, scaleFragment=TRUE, reScale=FALSE, 
    geneIdCol="transcripts_id")

# Build object that includes inron retention and exon-exon junction results
mzIntRefObjTmp<- mzIntRefObj[,c(3,4,7,8,11,12,1,2,5,6,9,10)]
library(DESeq2)
mzIntRefObjTmpInt<- mzIntRefObjTmp[rowData(mzIntRefObjTmp)$int_ex=="intron",]
meanFpkm<-apply(scaledRetention(mzIntRefObjTmpInt),2,mean)
meanFpkm
# One mutated sample feature very low FPKM and read counts hence it is removed
mzIntRefObjTmpFilSam<- mzIntRefObjTmp[,meanFpkm>500000]
mzExRefObjTmp<- mzExRefObj[,c(3,4,7,8,11,12,1,2,5,6,9,10)]
library(DESeq2)
mzExRefObjTmpFilSam<- mzExRefObjTmp[,meanFpkm>500000]

mzRefIntExSamFilObj<- interestResultIntEx(
    intObj=mzIntRefObjTmpFilSam, 
    exObj=mzExRefObjTmpFilSam, 
    intExCol="int_ex",
    mean.na.rm=TRUE, postExName="ex_junc" )
save(mzRefIntExSamFilObj,
    file="./mzRefIntExSamFilObj.rda")

# differential analysis
mzSamFilDiff<- deseqInterest(mzRefIntExSamFilObj,  
    design=~test_ctrl+test_ctrl:intronExon, 
    sizeFactor=rep(1,nrow(colData(mzRefIntExSamFilObj))), 
    contrast=list("test_ctrltest.intronExonintron",
        "test_ctrlctrl.intronExonintron"),
    bpparam = SnowParam(workers=50))
save(mzSamFilDiff, 
    file="./mzSamFilDiff.rda")

# Normalize counts for intron and save
mzIntRefObjSamFil<- 
    mzRefIntExSamFilObj[,
        colData(mzRefIntExSamFilObj)$intronExon=="intron"]
mzIntRefObjSamFilInt<- mzIntRefObjSamFil[
    rowData(mzIntRefObjSamFil)$int_ex=="intron",] 
library(DESeq2)
rlogMzIntRefObjSamFil<- rowMeans(rlog(as.matrix(counts(mzIntRefObjSamFilInt))))
save(rlogMzIntRefObjSamFil, file="./rlogMzIntRefObjSamFil.rda")
```

The next script shows the number and the fraction (%) of various types of significantly retained introns (when RGH3 gene is mutated):

```
load("./mzSamFilDiff.rda")
load("./mzRefIntExObj.rda")

# Filter sample with low normalized read counts
mzIntRefObjTmpInt<- mzRefIntExObj[,colData(mzRefIntExObj)$intronExon=="intron"]
meanFpkm<-apply(scaledRetention(mzIntRefObjTmpInt),2,mean)
meanFpkm
```

```
## SRR1282041 SRR1282042 SRR1282045 SRR1282046 SRR1282049 SRR1282050 
##   554268.5   568288.5   579858.0   525227.5   565276.1   257973.2 
## SRR1282039 SRR1282040 SRR1282043 SRR1282044 SRR1282047 SRR1282048 
##   612936.3   564129.6   475415.5   574259.9   571538.3   573359.4
```

```
mzIntRefObjTmpFilSam<- mzIntRefObjTmp[,meanFpkm>400000]
# P-values threshold definition
pThreshold<- 0.01
# Extracting Upregulated IR indexes 
mzSamFilUpInd<- which(mzSamFilDiff$padj<pThreshold & 
    mzSamFilDiff$log2FoldChange>0)
# Number of various significantly higher retained intron
table(rowData(mzRefIntExSamFilObj)$intron_type[mzSamFilUpInd])
```

```
## 
##    U12 U12/U2     U2 
##     61      0    709
```

```
# Number of all introns
table(rowData(mzRefIntExSamFilObj)$intron_type)
```

```
## 
##    U12 U12/U2     U2 
##    132      5 151828
```

```
# Share (%) of significantly retained IR from each type of intron
100*table(rowData(mzRefIntExSamFilObj)$intron_type[mzSamFilUpInd])/
    table(rowData(mzRefIntExSamFilObj)$intron_type)
```

```
## 
##        U12     U12/U2         U2 
## 46.2121212  0.0000000  0.4669758
```

Here we generate a plot similar to Fig.2 for the Maize data.

```
#Define function to draw curly braces
CurlyBraces <- function(x0, x1, y0, y1, pos = 1, direction = 1, depth = 1) {

    a=c(1,2,3,48,50)    # set flexion point for spline
    b=c(0,.2,.28,.7,.8) # set depth for spline flexion point

    curve = spline(a, b, n = 50, method = "natural")$y * depth

    curve = c(curve,rev(curve))

    if (pos == 1){
        a_sequence = seq(x0,x1,length=100)
        b_sequence = seq(y0,y1,length=100)  
    }
    if (pos == 2){
        b_sequence = seq(x0,x1,length=100)
        a_sequence = seq(y0,y1,length=100)      
    }

    # direction
    if(direction==1)
        a_sequence = a_sequence+curve
    if(direction==2)
        a_sequence = a_sequence-curve

    # pos
    if(pos==1)
        lines(a_sequence,b_sequence, lwd=1.5,   xpd=NA) # vertical
    if(pos==2)
        lines(b_sequence,a_sequence, lwd=1.5, xpd=NA) # horizontal

}

# Plotting data preparation
load("./mzRefIntExSamFilObj.rda")
load("./rlogMzIntRefObjSamFil.rda")

mzIntRefObjSamFil<- 
    mzRefIntExSamFilObj[,
        colData(mzRefIntExSamFilObj)$intronExon=="intron"]
mzIntRefObjSamFilInt<- mzIntRefObjSamFil[
    rowData(mzIntRefObjSamFil)$int_ex=="intron",] 
library(DESeq2)

# Get maximum average normalized IR counts of introns across each transcript 

maxRetTrMzSamFil<- tapply(rlogMzIntRefObjSamFil, 
    as.character(rowData(mzIntRefObjSamFilInt)[,"collapsed_transcripts_id"]), 
    max)
inTrSamFil<- names(maxRetTrMzSamFil)[which(as.numeric(maxRetTrMzSamFil)>0)]
mzIntFilSamFilBool<- 
    rowData(mzIntRefObjSamFilInt)[,"collapsed_transcripts_id"] %in% inTrSamFil
mzIntRefObjTmpSamFilRowFil<- mzIntRefObjSamFilInt[mzIntFilSamFilBool,]
mzRefIntExSamFilObjRowFil<- mzRefIntExSamFilObj[mzIntFilSamFilBool,]


par(lwd=1)
par(cex=1.5)
par(cex.axis=1.5)
par(cex.lab=1.5)
par(mfrow=c(4,1))
par(mar=c(5,5,2,1))
u12BoxplotNb(mzIntRefObjTmpSamFilRowFil, sampleAnnoCol="norm_rg3", lasNames=1,
    intExCol="int_ex", intTypeCol="intron_type", intronExon="intron", 
    boxplotNames=c(), outline=FALSE, plotLegend=TRUE, 
    geneIdCol="collapsed_transcripts_id", xLegend="topleft", 
    col=c("pink", "lightblue"), ylim=c(0,7000000), 
    ylab="FPKM", cex.axis=1.5, cex.lab=1.5, cexLegend=1.5, addGrid=TRUE,
    xaxt='n')
axis(1, at=c(6, 18.5), 
    labels=c("U2-type intron", "U12-type intron"))
title(main = "(A)", cex.main=1.5)
par(mar=c(6.5,5,2,1))
u12Boxplot(mzIntRefObjTmpSamFilRowFil, sampleAnnoCol="norm_rg3", 
    intExCol="int_ex",  intTypeCol="intron_type", 
    col=rep(c("orange", "yellow"),2) ,  lasNames=1, 
    outline=FALSE, ylab="FPKM", cex.axis=1.5, intronExon="intron", 
    addGrid=TRUE, boxplotNames= rep(c("U12","U2"), 2))
c(rep("RGH3mut",2), 
        rep("RGH3wt",2))
```

```
## [1] "RGH3mut" "RGH3mut" "RGH3wt"  "RGH3wt"
```

```
CurlyBraces(.7,2.3,-700000,-700000, pos = 2, direction = 2, depth=70000)
CurlyBraces(3.7,5.3,-700000,-700000, pos = 2, direction = 2, depth=70000)

mtext(c("RGH3mut", "RGH3wt"), at=c(1.5, 4.5), side=1, 
    line=3.2, cex=1.1)
title(main = "(B)", cex.main=1.5)
par(mar=c(5,5,2,1))
u12DensityPlotIntron(mzIntRefObjTmpSamFilRowFil, 
    type= c("U12", "U2UpDn", "U2Rand"), 
    fcType= "edgeR", sampleAnnoCol="test_ctrl", 
    sampleAnnotation=c("ctrl","test"), intExCol="int_ex", 
    intTypeCol="intron_type", strandCol= "strand", 
    geneIdCol= "collapsed_transcripts_id", naUnstrand=FALSE, col=c(2,5,6),
    lty=c(1,4,5), lwd=1, plotLegend=TRUE, cexLegend=1.5, 
    xLegend="topright", yLegend=NULL, 
    legend= c("U12-type intron (n=114)", 
        "U2-type up/down stream introns (n=228)",
        "U2-type random introns (n=114)"), 
    randomSeed=10,
    ylim=c(0,1), xlab=expression("log"[2]*" fold change FPKM"))
```

```
## Design matrix not provided. Switch to the classic mode.
```

```
title(main = "(C)", cex.main=1.5)
# psi plot

psiMz<-psi(x=mzRefIntExSamFilObjRowFil, 
    intCol= which(colData(mzRefIntExSamFilObjRowFil)$intronExon=="intron"), 
    exCol=which(colData(mzRefIntExSamFilObjRowFil)$intronExon=="exon"))
psiMzTest<- psiMz[,which(colData(mzRefIntExSamFilObjRowFil)$test_ctrl[
        colData(mzRefIntExSamFilObjRowFil)$intronExon=="intron"]=="test")]
psiMzCtrl<- psiMz[,which(colData(mzRefIntExSamFilObjRowFil)$test_ctrl[
        colData(mzRefIntExSamFilObjRowFil)$intronExon=="intron"]=="ctrl")]
difPsiAll<- (rowMeans(psiMzTest)-rowMeans(psiMzCtrl))
u12Ind<- u12Index(mzRefIntExSamFilObjRowFil, intExCol="int_ex", 
    intTypeCol="intron_type")
plot(density(difPsiAll[-u12Ind], na.rm=TRUE), type='l', lwd=1, lty=2, main="",
    xlab="ΔΨ")
points(density(difPsiAll[u12Ind], na.rm=TRUE), type='l', lwd=1, lty=1, 
    col="red")
text(x=0.05, y = 20, labels="U2", col="black")
text(x=0.4, y = 3, labels="U12", col="red")
title(main = "(D)", cex.main=1.5)
```

**Fig. S7.** FPKM-scaled retention levels of U12-type and U2-type introns across various samples in Maize data, excluding transcripts that feature only introns with low average read counts (i.e. 1 read or less). (A) Boxplot showing FPKM-scaled retention levels of the U12-type introns (middle) as compared to their (up/down)stream introns. The thick horizontal lines in boxplots represents the median values and the whiskers represent 1.5 times the interquartile range. The box extends from the first quartile to the third quartile. (B) Boxplot showing the distribution of the FPKM-scaled retention levels of U12-type introns compared to the U2-type introns in RGH3mut, and RGH3wt samples. (C) Density plot illustrating the frequency of the fold change (log2) of the retention levels of U12-type introns, randomly picked U2-type introns, and U2-type introns (up/down)stream of the U12-type introns when comparing RGH3mut to the RGH3wt samples of the Maize data. (D) Density plot illustrating the distribution of the frequency of the ΔΨ values (increase of fraction of spliced in) of the U12 and U2 type introns when comparing RGH3mut to the RGH3wt samples.

# Finally

The following scripts were used in R to generate the `suppl1.html` file from the `suppl1.Rmd` file.

```
library("rmarkdown")
render("./suppl1.Rmd")
```
